# Variable vertical land motion and its impacts on sea level rise projections

**DOI:** 10.1126/sciadv.ads8163

**Published:** 2025-01-29

**Authors:** Marin Govorcin, David P. S. Bekaert, Benjamin D. Hamlington, Simran S. Sangha, William Sweet

**Affiliations:** ^1^ Jet Propulsion Laboratory, California Institute of Technology, Pasadena, CA, USA.; ^2^ NOAA/National Ocean Service, Silver Spring, MD, USA.

## Abstract

Coastal vertical land motion (VLM), including uplift and subsidence, can greatly alter relative sea level projections and flood mitigations plans. Yet, current projection frameworks, such as the IPCC Sixth Assessment Report, often underestimate VLM by relying on regional linear estimates. Using high-resolution (90-meter) satellite data from 2015 to 2023, we provide local VLM estimates for California and assess their contribution to sea level rise both now and in future. Our findings reveal that regional estimates substantially understate sea level rise in parts of San Francisco and Los Angeles, projecting more than double the expected rise by 2050. Additionally, temporally variable (nonlinear) VLM, driven by factors such as hydrocarbon and groundwater extraction, can increase uncertainties in 2050 projections by up to 0.4 meters in certain areas of Los Angeles and San Diego. This study highlights the critical need to include local VLM and its uncertainties in sea level rise assessments to improve coastal management and ensure effective adaptation efforts.

## Teaser

Local land motion, often overlooked, can greatly underestimate sea level rise projections by 2050.

## INTRODUCTION

Relative sea level rise [RSLR; (*1*)] both now and in the future results from a combination of the rising oceans and the sinking of land. The relative contributions from the movement of land and ocean to sea level rise vary spatially due to the combined effect of several physical processes. These processes and their behavior in the past and present have been discussed and documented extensively in recent literature (*2*, *3*). Projecting contributions into the future, however, presents challenges closely linked to the maturity of the understanding of the individual processes (*4*). Accurate projections of future changes in sea level in relation to land are contingent upon a robust understanding of the individual processes that contribute to sea level change. The Sixth Assessment Report (AR6) from the Intergovernmental Panel on Climate Change (IPCC), relying on scientific progress in the preceding years, marked a considerable advancement in the field of relative sea level projections (*3*, *5*). The AR6 advanced upon previous assessment reports in its improved modeling of sterodynamic changes—that is, combined thermal expansion and ocean dynamic changes—enhanced knowledge of the behavior of ice sheets and an improved understanding of potential future contributions of glaciers and terrestrial water storage on sea levels. Together, these advances led to a substantial step forward in the modeling and projection of increasing sea level heights.

The approach for projecting coastal vertical land motion (VLM), which refers to the combined contribution from subsidence and uplift, however, remained largely unchanged between previous assessments and the AR6. The AR6 relied heavily on measurements of relative sea level change provided by long tide gauge records to indirectly infer the rate of VLM. This rate was then extrapolated linearly into the future (*3*, *6*, *7*), resulting in highly certain projections of VLM that were potentially missing important local information especially where no tide gauge is present. VLM is caused by various factors, including tectonic activities, glacial isostatic adjustment, sediment compaction, and the extraction of groundwater and other natural resources (*8*). In addition, VLM can be accompanied by additional land-surface changes related to sediment deposition and erosion processes (*9*). The result is considerable spatial and temporal variations in VLM arising from the complex interplay of these contributing factors. This poses challenges for accurately quantifying the impact on future sea levels.

Integration of direct observations of VLM presents a near-term path forward for improving projections of VLM contributions to RSLR. Global Navigation Satellite System (GNSS) stations provide precise measurements of land movement and can detect both the slow, steady movements of land and sudden shifts due to short-term processes. However, coverage (*10*) is uneven globally, and, even in areas with dense GNSS coverage, there remain large gaps that miss areas with large magnitude VLM [e.g., (*11*–*13*)]. Interferometric Synthetic Aperture Radar (InSAR), on the other hand, provides high–spatial resolution (*14*, *15*) estimates of VLM (as combined land-surface change from both deep and shallow processes) over large areas that can fill these gaps but requires GNSS as tie-in points for its placement in a common reference frame (*15*). Geodetic observations from both GNSS and InSAR are limited by record length extending back 2 to 3 decades (*16*, *17*). Nevertheless, the opportunity to use InSAR in combination with GNSS to improve upon the spatial and temporal information contained within relative sea level projections represents a potential step change of advancement for existing projections.

Here, we address challenges associated with adequately representing the rate of VLM over the time and space scales over which it varies and the resulting implications for projecting RSLR into the future. Specifically, we focus on the coasts of California, along the west coast of the United States, a region affected by several processes that lead to variations in VLM across a range of scales. From our analysis, we provide a path forward for producing improved projections of VLM that better capture the state of understanding of VLM on a local level that is potentially applicable to all coastal locations.

## RESULTS

### Spatially and temporally varying VLM

**Fig. 1.**
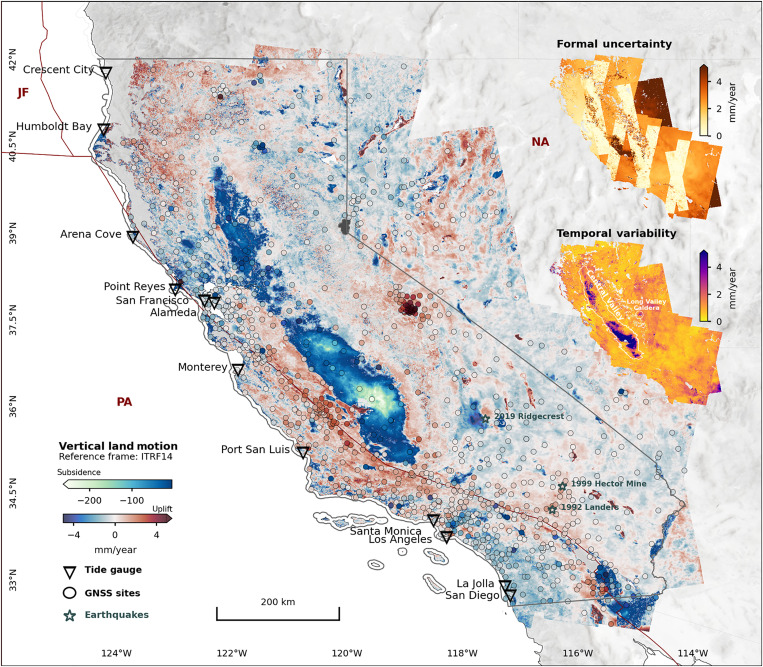
VLM with uncertainties. VLM (mm/year) for 2015–2023 estimated from Sentinel-1 InSAR in combination with GNSS data (InSAR-VLM), relative to ITRF2014 [International Terrestrial Reference Frame, 2014 solution; (*59*)]. Negative VLM values reflect subsidence, while positive values reflect uplift. Uncertainties on rates (formal uncertainties, 1σ) and its trend temporal variability (also in fig. S1) within observation period are shown as insets. Site/event locations are marked as GNSS (circles), tide gauge (inverted triangles), and major earthquakes (stars). Tectonic plate boundaries (maroon lines) follow: PA, Pacific plate; NA, North America plate; and JF, Juan de Fuca plate (*66*).

We characterize near-decadal (2015–2023) spatially varying VLM, across the state of California by combining high-resolution (90-m) InSAR from Sentinel-1 with GNSS data (Fig. 1; see Materials and Methods). Besides propagating uncertainties from both techniques (formal uncertainties in Fig. 1), we derive a temporal variability metric associated with our InSAR-VLM to separate temporally varying (nonlinear) processes from the stable (linear) ones. We calculate a temporal variability metric as a measure of the agreement between the full-record trend and the trends from different shorter segments (length of >3 years) of that record (see Materials and Methods). High temporal variability with respect to its surroundings indicates locations with notable trend fluctuations, while low temporal variability suggests constant trends throughout the observation period. This analysis advances the state of high-resolution VLM observations for the purpose of improving relative sea level projections, as previous studies (*14*, *18*–*21*) neglected internal variability and based their analysis on the data modeled solely as linear processes, despite the evidence to the contrary (*13*, *22*).

On a regional scale, our InSAR-VLM confirms a known widespread tectonic-induced land uplift (1 to 3 mm/year) in northern CA [associated with the Cascadia subduction zone; (*18*, *23*, *24*)] and subsidence around San Francisco and San Diego [associated with the San Andreas Fault System; (*18*, *24*, *25*)] (Fig. 1). We see post-seismic uplift around the 1999 Hector Mine, 1992 Landers (*24*), and uplift and subsidence associated with 2019 Ridgecrest earthquake (*26*). Most of these large-scale natural processes appear to be stable in this study time span (temporal variability < 2.5 mm/year, using 95 percentiles of data in fig. S1). Exceptions are time-dependent processes, such as the episodic deflation-inflation events at Long Valley Caldera (*27*) (temporal variability of 2 to 4 mm/year). Furthermore, processes related to extraction and injection of hydrocarbon and groundwater resources as well as recharge of aquifers lead to variable spatial and temporal surface responses often correlated with pronounced human activities and climatic events [precipitation and drought periods; (*28*)]. Here, this appears evident in very high temporal variability across the fast-subsiding Central Valley (VLM < −200 mm/year with temporal variability from 3 to 350 mm/year; Fig. 1). Similar temporal trend variations (temporal variability of 3 to 5 mm/year), notably elevated in their surroundings, are observed over other aquifers, e.g., Santa Clara in the San Francisco Bay area, Santa Ana in Los Angeles, and Chula Vista in San Diego (fig. S1).

**Fig. 2.**
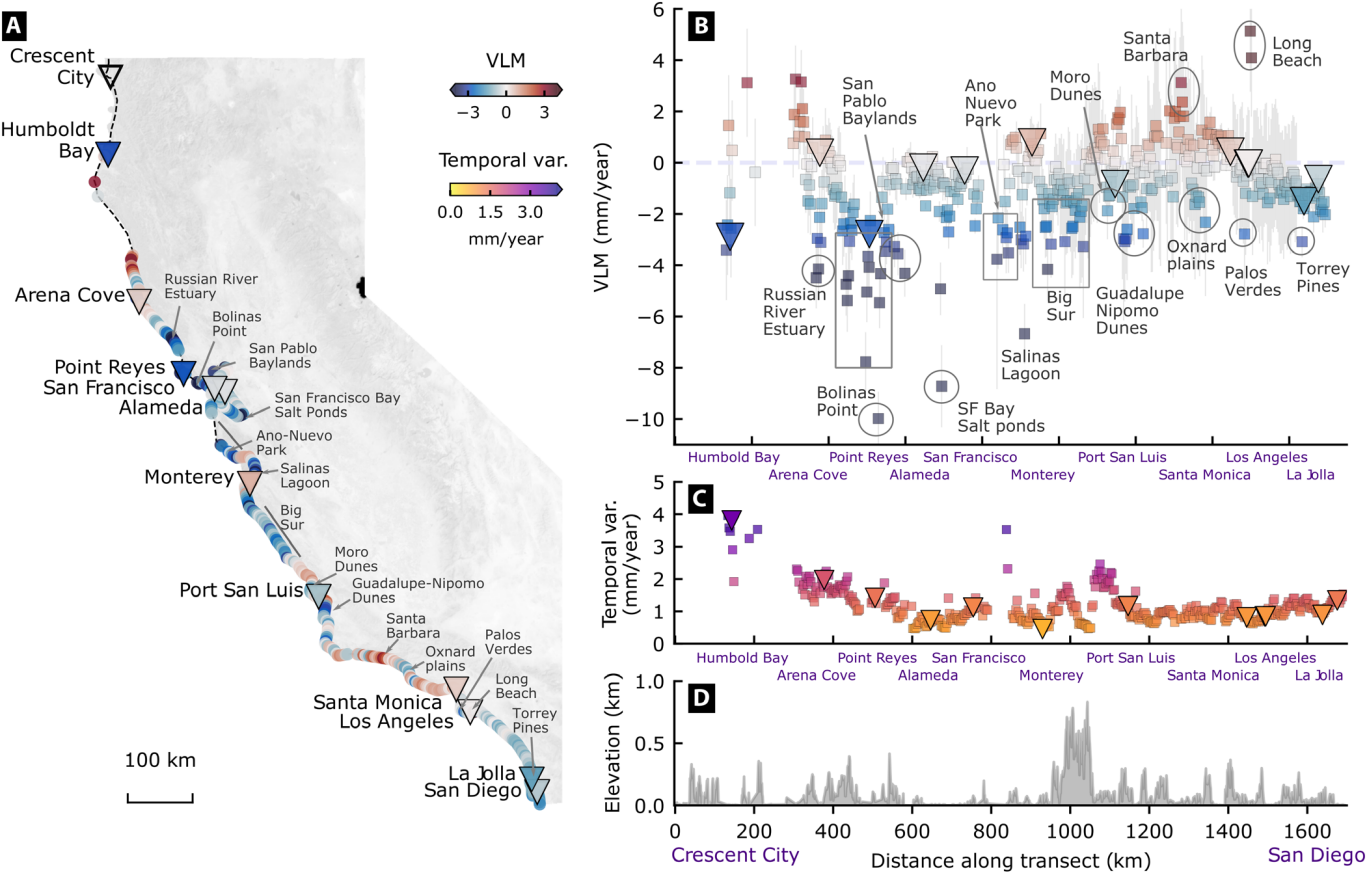
Variable VLM along the California coasts, with hot spots. (**A**) Overview of InSAR-VLM at tide-gauge locations (inverted triangles, mean of data within 0.5 km around), and mean estimates (square markers) sampled every 3 km along the 1700-km-long coastline, (**B** and **C**) transect of InSAR-VLM from (A) with associated 1σ formal uncertainties (gray lines around square markers), and temporal variability (C) over the (**D**) elevation [Copernicus 30m Digital Elevation Model; (*67*)]. Mean rates are weighted by associated formal uncertainties and temporal variability within the sample size.

Within these regional patterns, the high-resolution InSAR-VLM reveals spatial varying and localized motion along the 1700-km California coastline (Fig. 2). We find local zones of downward motion, associated with slow-moving landslides along the rugged coastal terrain [e.g., Big Sur mountains and Palos Verdes Peninsula; (*29*)], eroding cliffs of the Torrey Pines State Park (*30*) (preceding cliff collapse at the Black Beach in January 2023), and the retreating beaches of Morro and Guadalupe Nipomo Dunes [where restoration projects are ongoing; (*31*)]. We also see high rates (VLM < −4 mm/year, with temporal variability > 3 mm/year) over marshlands in San Pablo Baylands, Ano Nuevo State Park, and the surrounding Salinas River Lagoon and Sonoma County’s Russian River Estuary. These rates and their variability are likely influenced by vertical accretion and/or erosion (*8*, *9*). Uplift hot spots (>3 mm/year) are evident in Long Beach where fluid extraction and injection take place (*32*, *33*) and over the Santa Barbara groundwater basin that has been steadily recharging since 2018 (*34*). Other areas related to human activities coincident with varying lands over San Francisco Salt Ponds [VLM rates from −25 to 10 mm/year, undergoing wetland restoration efforts; (*35*)], and the Oxnard Plain [VLM: −8 to 3 mm/year, temporal variability of 2 to 3 mm/year, known for saline intrusion in unconfined aquifers; (*36*)]. Passing the San Francisco Bay Area up north, we find an above average land subsidence (>−3 mm/year) consistent with the nearby GNSS [site PTRL: −4 ± 1 mm/year, Nevada Geodetic Laboratory (NGL), http://geodesy.unr.edu, accessed 5 June 2024; (*16*)] and that in previous study (*32*), stretching from Bolinas Point to Point Reyes. The localized features listed above highlight the existence of highly dynamic land in between tide gauges that act as connecting points between rising seas and moving lands. With rising seas, these locations are likely to be more exposed to waves, flooding, inundation, sediment loss, and saltwater intrusion (*37*, *38*).

We find that these localized features are consistent with previous studies across California [e.g., (*11*, *32*, *39*)] but differ somewhat from the findings of coastal InSAR VLM study by Blackwell *et al.* (*18*). For instance, Blackwell *et al.* (*18*) report subsidence rates of up to −3 mm/year for San Francisco Bay hot spots such as San Francisco International Airport, Foster City, and Bay Farm Island, while we observe rates exceeding −5 mm/year, consistent with other studies (*11*, *32*). Furthermore, we find localized land motion near the Palos Verdes, Long Beach, and Santa Monica fault zone in Los Angeles, as well as Torrey Pines State Park and Chula Vista in San Diego, which are in line with other studies (*32*, *39*) but are not documented by Blackwell *et al.* (*18*). One possible explanation is post-processing filtering and interpolation in (*18*), which may have reduced the effective data resolution of their results.

### Projection of VLM contribution to relative sea level

**Fig. 3.**
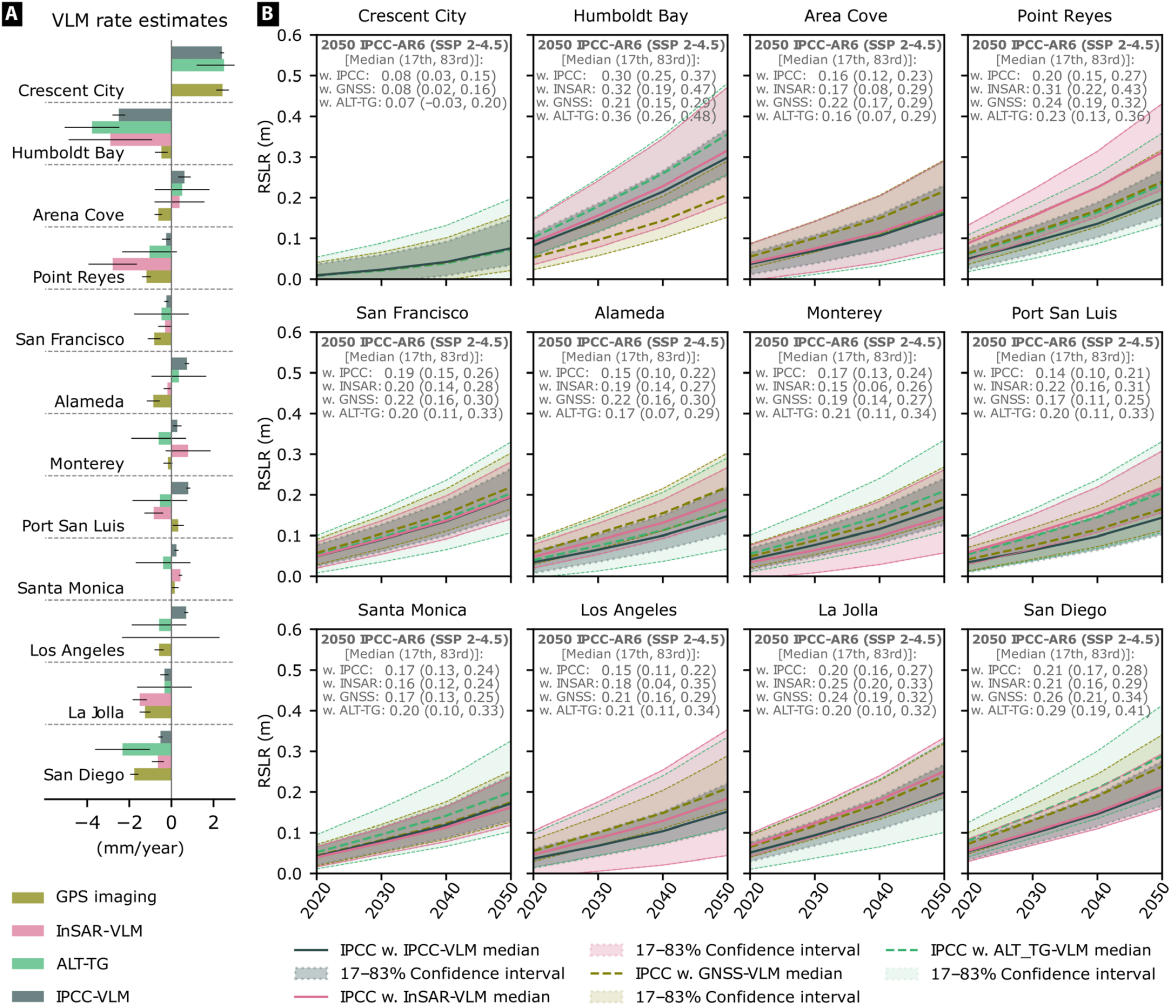
VLM for regional relative sea level projections. VLM contribution to near-term (2020–2050) regional relative sea level projections for California: (**A**) comparison of InSAR-VLM with other contemporary [GPS imaging; (*10*), ALT-TG; (*41*)], and IPCC-VLM (*6*) estimates at tide gauges, with their 1σ uncertainty bounds; (**B**) contribution of different VLM estimates and their uncertainties to regional projections in 2050 [median with uncertainties defined by 17th to 83rd percentile bounds; (*7*)]. VLM negative and positive trends from (A) are reversed to determine its contribution to relative sea level in the SSP2-4.5 medium confidence scenario (*68*).

The IPCC AR6 framework (*3*) relies on historical data (geological and model rates) interpolated on tide-gauge locations for VLM contributions (i.e., regional estimates) to relative sea level projections [IPCC-VLM; (*6*, *7*)]. The results can be at odds with estimates of contemporary (1993 to present) VLM derived either from InSAR (*14*, *21*), nearby GNSS sites (*10*, *22*), or indirectly by subtracting gridded altimetry from tide-gauge data (*40*, *41*). In Fig. 3, we analyze VLM for regional projections obtained at tide gauges by comparing our high-resolution InSAR-VLM rates with the IPCC-VLM and other present-time estimates from the GPS imaging (*10*) and 1° ALT-TG [altimetry minus tide gauge; (*41*)] methods. We find InSAR-VLM to agree well, within 0.5 mm/year, with the GPS imaging where contributing GNSS sites are not too far away (<2 km) from a tide gauge, as in the example of La Jolla (where GNSS is within 50 m). Increasing distances between GNSS and tide gauge tend to typically drive the GPS imaging to disagree with others, as found at the Humboldt Bay and Port San Luis (with closest GNSS sites > 5 km away; fig. S2). We see similar spatial limitations with ALT-TG estimates, where distance from shore to deep ocean likely influences the level of agreement with other methods due to coastal ocean variability (*42*). When this distance is within half of its resolution of ~100 km, as in northern California, we find it to agree well (<0.5 mm/year) with InSAR-VLM, specifically at Humboldt Bay, Port San Luis, Arena Cove, and San Francisco (fig. S2). Furthermore, different VLM trends at Arena Cove and Monterey (Fig. 3) are likely associated with time-dependent processes. Nearby GNSS sites [P059 and P231, NGL time series; http://geodesy.unr.edu, accessed 5 June 2024; (*16*)] show overall downward trends over the 2008–2024, which suddenly changed its course upward from 2016 to 2022, consistent with our InSAR-VLM. This points that local processes can cause VLM to differ from regional and historic rates, depending on the observation period and nature of underlying processes.

**Fig. 4.**
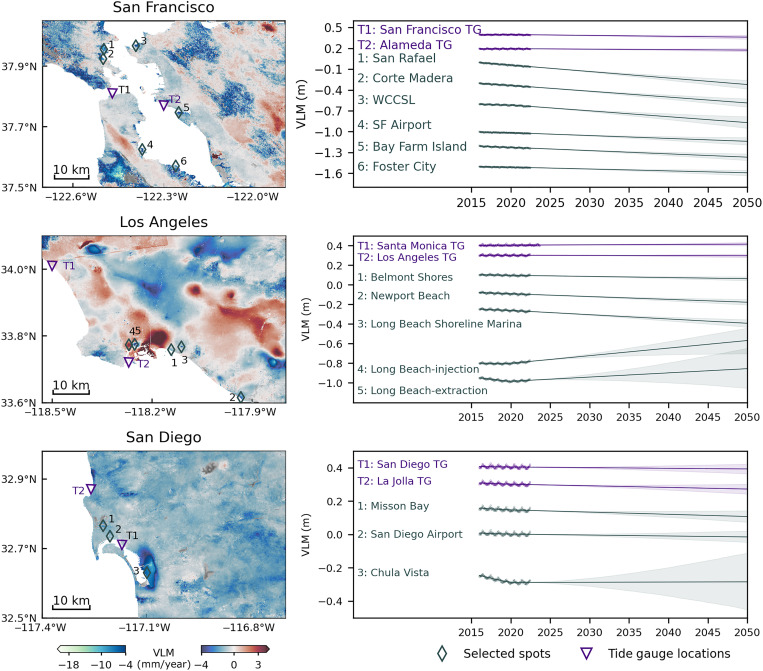
Local versus regional VLM projections in 2050. Differences between local (enumerated gray diamonds) and regional (at tide gauges, purple inverted triangles) VLM projections in 2050, using examples in urban centers of the San Francisco Bay area, Los Angeles, and San Diego. Local VLM is projected using reconstructed VLM time series (on the right), where linear and nonlinear processes are accounted for with probabilistic uncertainties in the interval of 17th to 83rd percentiles (see Materials and Methods). Time series and projections at selected spots are manually offset for visualization purposes.

At present, we find our InSAR VLM rates to contribute more (>1 mm/year) than IPCC-VLM to regional RSLR at La Jolla (−1.51 ± 0.34 mm/year versus −0.33 ± 0.21 mm/year), Port San Luis (−0.83 ± 0.45 mm/year versus 0.80 ± 0.10 mm/year), Alameda (−0.18 ± 0.18 mm/year versus 0.74 ± 0.10 mm/year), and Point Reyes (−2.78 ± 1.15 mm/year versus −0.25 ± 0.20 mm/year), based on the significance ratio (see fig. S3) from Pfeffer *et al.* (*43*). When projected to 2050, these locations could experience additional 0.03 to 0.08 m of RSLR than anticipated (Fig. 3). No meaningful difference from the IPCC-VLM is observed at Humboldt Bay, Arena Cove, San Francisco, Monterey, Santa Monica, and San Diego. Here, higher temporal and/or spatial VLM variability is accounted for in relative sea level projections with upper and lower bounds, as shown for the Los Angeles tide gauge near the hydrocarbon extraction fields in Long Beach (Figs. 2 and 4).

A key remaining question, however, is whether regional relative sea level projections derived at the tide gauges are representative in locations between tide gauges due to spatially and temporally varying VLM. In Fig. 4, we see how fine-resolution InSAR-VLM time series and their short-term projections can greatly differ within the low-lying (elevation < 10 m) highly populated areas as in San Francisco, Los Angeles, and San Diego. We find a steady high-rate subsidence (>10 mm/year) driven by sediment compaction, notably altering VLM contributions at street level in San Rafael, Corte Madera, Foster City, and Bay Farm Island. At short timescales, VLM here additionally increases RSLR by 0.07 to 0.26 m in 2050 and then found at the San Francisco and Alameda tide gauge (0.19 m in 2050). This indicates that, in some locations, regional estimates largely underestimate future sea level rise by more than a factor of two (e.g., regional RSLR of 0.19 m versus local of 0.45 m by 2050). Proportionally, this also accelerates the exposure of certain critical infrastructure to rising seas, as in the example of the San Francisco International Airport (RSLR: 0.29 m in 2050). Subsidence hot spots on reclaimed lands are also found in southern CA. In areas like Newport Beach (Newport Island) and Long Beach (Belmont Shores and Shoreline Marina) in Los Angeles, localized VLM adds 0.05 to 0.15 m on top of 0.17 m at nearby tide gauges in 2050. In San Diego, we found local land to raise RSLR in Mission Bay by 0.02 m above 0.24 m at La Jolla tide gauge. While some areas steadily outpace regional RSLR estimates, others undergo trend shifts influenced by human activities. For example, at the West Contra Costa Sanitary Landfill (WCCSL) in the Bay area, rate of subsidence slightly increased from August 2021. In Los Angeles, areas around injection and extraction Long Beach wells experienced trend shifts in February 2021, while, in San Diego, Chula Vista subsidence slowed down in October 2019. Because these trends vary temporarily, projections for the next three decades show much wider ranges of uncertainty (e.g., WCCSL, 0.17 m; Long Beach injection/extraction, 0.25/0.41 m; and Chula Vista, 0.34 m; see Fig. 4 and table S1) compared to other locations with stable linear trends (average uncertainty range, 0.07 m in 2050; Fig. 4 and table S1). To properly account for future trend changes in projections, close monitoring of these hot spots in fine detail is essential as the record continues to lengthen. Beside updating projections, InSAR-VLM time series can also assist in timely adaptation efforts, especially if VLM trends overly shift downward at certain times, leading to increased exposure to sea level rise.

## DISCUSSION

Along 1700 km of the California coast, we illustrate that VLM is neither constant nor uniform but varies at different spatial and temporal scales due to a combination of natural and human-induced factors. These processes can notably amplify local RSLR, sometimes more than doubling regional estimates, as found in the Los Angeles and San Francisco Bay, the two most populated areas in California. Therefore, including local VLM contributions in relative sea level projections can be crucial for near-term adaptation strategies. However, current frameworks [e.g., (*7*, *44*, *45*)] primarily rely on regional relative sea level projections, largely lacking data on local land motion and thus underestimating its impacts and direct socioeconomic consequences (*2*, *37*, *46*).

Our study demonstrates that InSAR combined with GNSS can effectively capture and enhance contemporary VLM contributions to both regional and local relative sea level projections. Further refinement may involve distinguishing contributions from deep from shallow land processes, which would require additional modeling beyond this study’s scope. When considering regional projections, direct InSAR measurements near tide gauges might be more representative than those inferred from GNSS sites located kilometers away. In addition, contemporary VLM near tide gauges, sometimes, differs from the historic trends used in the IPCC AR6 report. These differences could be indicative of local time-dependent (i.e., nonlinear) processes, as observed in Monterey.

Recent work by Oelsmann *et al.* (*22*) highlights the importance of addressing nonlinear VLM and its associated uncertainties in regional relative sea level projections, particularly in tectonically active and populated regions, like California. Here, we address nonlinearities in high-resolution InSAR-VLM rates with a temporal variability metric, bringing this type of analysis on a local level (Fig. 1; see Materials and Methods). This allows separating temporary varying from stable land processes in fine detail, particularly linked to human-induced activities that are identified as one of the challenges in sea level modeling (*2*). Furthermore, formal uncertainties tend to underestimate nonlinear motion due to incorrect model assumptions. Our study provides a path forward for high-resolution projections with realistic uncertainties, directly estimated from InSAR-VLM time series that capture the complete trend history (Fig. 4; see Materials and Methods). Once properly accounted for, we found that the uncertainty bounds for nonlinear processes can greatly exceed the regional RSLR estimates by 2050 (e.g., 0.34 m of VLM uncertainty bounds at Chula Vista versus RSLR of 0.19 m at San Diego tide gauge in 2050; Figs. 3 and 4). This demonstrates a low confidence in projecting nonlinear processes, even in the near-term future. Additionally, some natural processes, such as earthquakes or landslides, can induce sudden land motion on the order of meters or more, which is difficult, if not impossible, to account for in projections due to their unpredictable nature. Thus, VLM estimates here and their impacts on relative sea level can be only properly accounted for with further monitoring. We suggest translating this into a dynamic relative-sea level projection framework, where the VLM component in the relative sea level budget is regularly updated as high-resolution data become available.

In summary, our study underscores the importance of incorporating local VLM with realistic uncertainties into relative sea level projections to improve and ensure effective coastal adaptation strategies. Further work might be necessary to incorporate these data with local ocean processes to make the projections even more accurate, as noted in the IPCC AR6 report (*3*). The computation burden associated with high-resolution VLM represents a challenge in making these data available all around the globe and to be considered in the next IPCC framework. Nevertheless, we note the recent progress in this direction led by the European Ground Motion Service [https://egms.land.copernicus.eu/; e.g., (*47*)] and the Observational Products for End-Users from Remote Sensing Analysis (www.jpl.nasa.gov/go/opera) projects in bringing this type of data systematically over Europe and North America.

## MATERIALS AND METHODS

### InSAR and time-series analysis

We use open-access Sentinel-1 Geocoded Unwrapped Phase (S1-GUNW) products produced by the Advanced Rapid and Imaging Analysis (ARIA; https://aria.jpl.nasa.gov/) project at Jet Propulsion Laboratory (JPL) and California Institute of Technology to create a land-surface displacement map across the state of California. ARIA S1-GUNW products provide geocoded unwrapped interferograms at 90 m posting in the radar line of sight (LOS), with associated qualitative metrics (coherence and unwrapped phase connected components) included. Products are free and openly distributed through NASA’s Alaska Satellite Facility data archive. State-wise coverage was obtained by using 61.451 GUNW products over nine tracks (ascending and descending orbit geometries; table S2) between 2015 and 2023 (fig. S4). We use the open-access ARIA-tools software package (*12*) for preprocessing and the Miami INsar Time-series software in Python [MintPy; (*48*)] for time-series analysis. Each track is processed separately because of different satellite imaging geometry and relative nature of the unwrapped phase.

The GUNW products are unwrapped using Statistical-cost, Network-flow Algorithm for Phase Unwrapping [SNAPHU; (*49*)]. We developed a method to sequentially stitch multiple frames of unwrapped phase on the same acquisition date across the same track. This method was contributed to the open-source ARIA-tools software. As unwrapping is not trivial, unwrapping errors exist, manifesting as integer 2π phase jumps. Our sequential stitcher uses connected components, a metric provided by SNAPHU for identifying reliable unwrapped regions, to perform unwrapped phase corrections in the overlap regions between consecutive GUNW products using the phase. To minimize the unwrapping misalignments, the stitcher follows an iterative region-growing logic by adding 2π-integer corrections between overlapping components in forward and backward direction for adjacent frames. The result is merged unwrapped and its associated connected component product.

Unreliable regions (typically characterized by low coherence) of the merged unwrapped phase are masked out by using the zero connected component provided by SNAPHU (*49*). We use ARIA-tools for all the preprocessing steps: creating merged unwrapped phase, coherence, connected components, water mask, and imaging geometry as outputs, for subsequent time-series processing with MintPy. Using MintPy, we invert interferometric-pair network weighted by the interferogram phase variance (*50*) to obtain time-series LOS displacements with respect to arbitrary defined spatial and temporal reference. As the network precision depends on the interferograms quality, i.e., level of decorrelation and unwrapping errors, we first run the MintPy unwrapping error correction, phase bridging (*48*), to connect and correct isolated unwrapping errors, and then manually inspect and discard interferograms that remain noisy in each stack. Because of variability in land cover, topography, and climate across the state, we apply adaptive network modification where needed. We excluded dates from the inversion for pixels consistently decorrelated across all interferometric pairs, such as winter dates over snow-covered mountainous areas, dates with inundation from snowmelt or high tides, and similar conditions affecting satellite acquisitions. Network connectivity for these pixels is maintained using annual pairs from coherent seasons. Specifically, this mostly applies for the tracks covering northern California (fig. S5). We further reduce the noise by using tropospheric phase delay correction calculated with the ERA-5 global atmospheric model (*51*) and topographic residual correction as part of the MintPy workflow. Relative LOS displacement rates with their formal uncertainties (fig. S5) are estimated through least-squares parametric fitting to time series including for linear rate, semiannual and annual, and Heaviside step parameters (Eq. 1). Specifically, we add steps for 2019 M7.1 Ridgecrest, 2020 M6.5 Monte Cristo, and 2021 M6.0 Antelope Valley earthquakes (US Geological Survey, 2024, Earthquake List, www.usgs.gov/natural-hazards/earthquake-hazards, accessed 5 June 2024) to estimate for their coseismic step displacement through time-series fitting

$${\bar{x}}_{t}=a+r\left(t-t_{0}\right)+\sum_{j=1}^{\mathrm{nj}}b_{j}H\left(t-t_{j}\right)+\sum_{k=1}^{\mathrm{nf}}\left[s_{k}\text{sin}\left(\omega_{k}t\right)+c_{k}\text{cos}\left(\omega_{k}t\right)\right]$$ (1)

where *a* is intercept, *r* is constant trend, *H* is the Heaviside step function, *b* is direction and magnitude of step at time $t_{j},$ and *nj* is the number of steps. The $s_{k}$ and $c_{k}$ are the Fourier coefficients for the harmonic with angular frequency $\omega_{k}=2\pi /t_{k}$ for $t_{k}=1/k$ years, and nf represents the total number of frequencies or periods.

### Temporal variability

We perform trend variability analysis [adapted from (*10*, *52*) for high-resolution data] to examine the stability of estimated secular LOS displacement rates. Trend variability analysis is obtained by comparing the best fit trends to data from different moving windows with the trend estimate from the full data record. As most of our InSAR time series span 6 to 7 years, we start with a moving window of 3 years and then gradually increase it to 5 years by extending 1 year in each round. Moving windows are shifted forward by 1 month until the end of the time series, in each round. The temporal variability metric is quantified as the median absolute deviation (MAD) of the disagreement between moving window trends and a full-record trend (Eq. 2). Using MAD ensures additional robustness due to its lower sensitivity to outlier trends from moving windows (*10*). Besides capturing high variability associated with nonlinear anthropogenic processes, the analysis proves also to be a good indicator of unwrapping errors (fig. S1).

$$\text{MAD}\left(∆t\right)=\text{median}|r_{i}{\left(\Delta t\right)}_{\mathrm{im}}-{\bar{r}}_{t}|\;\text{for}\;i=3,4,\ldots ,t$$ (2)

where $r_{r}\left(∆t\right)$ is the rate from the moving window *i*, length ∆t (starting from 3 years length), with *m* step ahead of 0.083 years; and ${\bar{r}}_{t}$ is the rate from the time-series full record t, calculated through parametric fitting (Eq.1).

### GNSS model

We rely on GNSS data to calculate translation of relative InSAR LOS land-surface displacements into a standard geodetic reference frame, i.e., InSAR calibration. We choose to go with 24-hour GNSS position time-series solutions in International Terrestrial Reference Frame 2014 (ITRF14) realization provided by the NGL (*16*) due to higher number of sites in comparison to other providers in the area, its open access and standardized database with documented discontinuities associated with instrument changes and earthquakes. We use GNSS time-series data in between 2012 and 2023 to obtain representative decadal horizontal and vertical displacement trends spanning across the InSAR time series. Further, we select sites with minimum duration of 3 years (*53*) and gap percentage smaller than 35% of the record calculated using 1 day sampling. During manual screening of selected 1020 GNSS sites, we remove the discontinuity steps on the same date and the ones with no data in between to avoid design matrix singularity in parametric fitting and add steps where missing (table S3). We use the Hector package (*54*) for GNSS time-series analysis to estimate a linear trend with an assumed temporal correlated noise (power-law + white noise). Outliers based on criteria in (*55*) are removed before the trend estimation.

Although spatially sparse in comparison to InSAR, GNSS provides a more accurate estimate of very slow >50-km large-scale motion (*52*), e.g., due to plate motion, postglacial rebound, and tides. Previous studies (*56*) report planar ramps in InSAR displacement rates associated with large-scale geophysical (e.g., plate motion) processes that are exacerbated by moving away from its reference point due to its side-looking imaging geometry. As such, simple affine transformation [e.g., (*13*, *14*, *18*)] is not adequate for a translation of relative large-scale InSAR displacement into a GNSS reference frame. We develop a GNSS displacement rate model as a calibration plane to accommodate for this spatially variable alignment between GNSS and InSAR. We interpolate GNSS rates on a 25 km–by–25 km grid, with additional points along San Andreas Fault System, using an extended least-squares collocation [Hv-LSC; (*57*)] method that considers spatial correlation between horizontal displacement components with plate boundaries constraints. Before this, we first omit the sites with apparent localized motion based on 2-sigma criteria applied on trend variability metric (*d*_North-South_ > 0.30 mm/year, *d*_East-West_ > 0.28 mm/year, and *d*_VLM_ > 1 mm/year) and combined horizontal and vertical trend uncertainties ($\sigma_{3D}$ > 2 mm/year) and remaining high-rate vertical rates (VLM > 3 mm/year). Second, we model and remove Pacific and North America plate motion using ITRF14 absolute plate rotation poles from Altamimi *et al.* (*58*) to remove trends that could affect empirical covariance estimation. As plate models do not capture elastic motion near plate boundaries, we artificially increase the distances by 1500 km between sites on different plates. We use Gauss-Markov first-order covariance function model in empirical covariance analysis to determine signal *c*_0_ variance and *d*_0_ correlation length for each horizontal and vertical displacement rate component assumed to be trend-free. However, a near-field signal along the San Andreas fault system (fig. S5) requires additional modeling that was out of the scope of this study. We observe through trial and error that the remaining trend signal only affects interpolation uncertainties and not its values. Therefore, to obtain more realistic interpolation uncertainties, we use correlation function, starting covariances with mean rate uncertainties estimated prior with a Hector software (*54*) and moving variance approach within radius of 850 km to adapt to the non-stationary residual horizontal field (*57*). The final stochastic parameters for the collocation process are as follows: horizontal, *c*_0_ = 0.2 mm^2^/year^2^ and *d*_0_ = 225 km; and vertical, *c*_0_ = 0.4 mm^2^/year^2^ and *d*_0_ = 52 km. The removed trends associated with Pacific and North America plate motion were restored with estimated model value on interpolation grid points. The final model (fig. S6) precision of 0.1 mm/year (North), 0.1 mm/year (East), and 0.2 mm/year (up) is obtained through model comparison with initial sites displacement values.

### InSAR referencing to ITRF14

As our GNSS model captures long-wavelength signals, we use simple bilinear interpolation to sample horizontal and vertical model rates onto the InSAR grid. We then project the model rates to each InSAR track LOS to subtract the projected model from the InSAR rates. The obtained residual plane is used as a base to estimate the calibration surface. First, we omit the high residual values associated with a high-rate subsidence observed in the Central Valley using 2ℴ criteria. Second, we interpolate the introduced gaps to ensure a spatially continuous residual surface. Further, we develop a moving window smoothing algorithm using weighted bilinear planar fitting that acts as a low-pass filter. The smoothing windows adapt to the valid InSAR data length, which is skewed because of satellite near-polar orbit trajectory. Each window is expanded by its size in all directions to avoid edge artifacts. We select the window size to be 25 km by 25 km to match the resolution of the GNSS model. We use the GNSS model and InSAR measurement uncertainties as weights in a least-squares inversion for the best fit planar parameters.

The low-passed smoothed residual surface is then modeled with the estimated parameters to the original window size with its uncertainties. This allows a full error propagation from InSAR measurement, GNSS model, and smoothing procedure uncertainties (Eq. 3). The obtained low-pass residual surface translates relative InSAR displacements to the GNSS plane that is positioned in a geodetic reference frame. We assume that the residual surface captures long-wavelength (>30 km) geophysical signals and untreated ionospheric noise in InSAR rates. We apply the latter to all InSAR tracks to obtain calibrated InSAR LOS rates (fig. S7) in the ITRF14 reference frame (*59*)

$$\sigma_{{\text{InSAR}}_{\text{calibrated}}}=\sqrt{\sigma_{\text{InSAR}}^{2}+\sigma_{\text{GNSS}}^{2}+\sigma_{\text{bilinear model}}^{2}}$$ (3)

### LOS decomposition for three-dimensional land motion

InSAR measures land motion in its LOS direction, defined by satellite imaging incidence angle and trajectory heading. As such, one-dimensional LOS observations capture both projected real horizontal and vertical motion along this displacement vector. This leaves us with an ill-conditioned linear system of equations with three unknowns (Eq. 4), which we solve with least-squares adjustment by using calibrated LOS observations from different satellite imaging viewing angles, e.g., ascending (East-looking) and descending (West-looking) orbit, with additional North-South motion from the GNSS data (Eq. 5). Here, we use all “stable” GNSS sites preselected for the GNSS model to obtain interpolated horizontal motion on the InSAR grid with Hv-LSC method (*57*). In the case of only one viewing geometry, we constrain horizontal motion using East-West and North-South GNSS displacements. We note that any localized horizontal land motion, not captured by the resolution of the input GNSS data, might lead to overestimation or underestimation of decomposed values, e.g., in case of landslides or subsidence radial deformation. The amount of over- or underestimation relates to amplitudes of missed East-West and North-South displacements and INSAR measurement sensitivity to those displacement components (absolute average displacement unit vector $\left[u_{\text{ns}}\;u_{\text{ew}}\;u_{\text{vlm}}\right]$ values for Sentinel-1: $u_{\text{ew}}$, ~0.63; $u_{\text{ns}}$, ~0.11; and $u_{\text{vlm}}$, ~0.78). InSAR measurement is least sensitive to North-South motion due to its side-looking geometry and near-polar orbits.

$${\text{dInSAR}}_{\text{LOS}}=\left[u_{\text{ns}},\;u_{\text{ew}},\;u_{\text{vlm}}\right]*{\left[d\text{NS},\;d\text{EW},\;d\text{VLM}\right]}^{T}$$ (4)

where $u_{\text{ns}}=\text{cos}\left(\alpha \right)*\text{sin}\left(\phi \right),u_{\text{ew}}=-1*\text{sin}\left(\alpha \right)*\text{sin}\left(\phi \right),\;\text{and}\;u_{\text{vlm}}=\text{cos}\left(\phi \right)$. α is satellite orbit heading angle that represents the vector from the target to sensor and is measured from the north in the counterclockwise direction and ϕ is the incidence angle of the radar imaging vector measured from vertical at the target. *d*NS and *d*EW is horizontal land motion in North-South and East-West direction, respectively, and *d*VLM is vertical land motion

$$x_{3x1}=(A_{3\mathrm{xm}}^{T},P_{\mathrm{mxm}},A_{\mathrm{mx}3})*(A_{3\mathrm{xm}}^{T},P_{\mathrm{mxm}},l_{\mathrm{mx}1})$$ (5)

where

$$P_{\mathrm{mxm}}=\text{diag}\left[1/\sigma_{I{\text{nSAR}}_{1}}^{2},1/\sigma_{I{\text{nSAR}}_{2}}^{2},\ldots 1/\sigma_{I{\text{nSAR}}_{n}}^{2},1/\sigma_{{\text{GNSS}}_{\text{ns}},}^{2}h/\sigma_{{\text{GNSS}}_{\text{ew}}}^{2}\right]$$

$$A_{{\text{InSAR}}_{\mathrm{nx}3}}=\left[\begin{array}{ccc} u_{{\text{ns}}_{1}} & u_{{\text{ew}}_{1}} & u_{{\text{vlm}}_{1}}\\ u_{{\mathrm{ns}}_{2}} & u_{{\text{ew}}_{2}} & u_{{\text{vlm}}_{2}}\\ \vdots & \vdots & \vdots \\ u_{{\mathrm{ns}}_{n}} & u_{{\text{ew}}_{n}} & u_{{\text{vlm}}_{n}} \end{array}\right],\;\;A_{{\text{GNSS}}_{\text{ns}}}=\left[\begin{array}{ccc} 1 & 0 & 0 \end{array}\right],\;\;A_{{\text{GNSS}}_{\text{ew}}}=\left[\begin{array}{ccc} 0 & 1 & 0 \end{array}\right]$$

$$A_{\mathrm{mx}3}={\left[\begin{array}{ccc} A_{{\text{InSAR}}_{\mathrm{nx}3}} & A_{{\text{GNSS}}_{\text{ns}}} & A_{{\text{GNSS}}_{\text{ew}}} \end{array}\right]}^{T}$$

$$l_{\mathrm{mx}1}={\left[d{\text{InSAR}}_{1}\;d{\text{InSAR}}_{2}\ldots d{\text{InSAR}}_{n}\;d{\text{GNSS}}_{\text{ns}}\;d{\text{GNSS}}_{\text{ew}}\right]}^{T}$$

$$m=n*\;\text{InSAR}+{\text{GNSS}}_{\text{ns}}+h*{\text{GNSS}}_{\text{ew}}$$

$$h=\left\{\begin{array}{cc} 0 & \text{if}\forall {\text{InSAR}}_{1,n}∋\text{ascending}\wedge \text{descending}\\ 1 & \text{else} \end{array}\right.$$

*A* is a design matrix, *P* is a weight matrix, *l* is a vector of observations, and *x* is a vector of unknowns (*d*NS, *d*EW, and *d*VLM). *n* is the number of InSAR datasets with different imaging geometries (tracks) used in the LOS decomposition.

Formal uncertainties associated with decomposition results (North-South, East-West, and VLM) and obtained with Eq. 6

$$\text{mse}=\frac{{\left(l-\mathrm{Ax}\right)}^{T}P\left(l-\mathrm{Ax}\right)}{m-3},\;\;Q_{\mathrm{xx}}=\text{mse}*\left(A^{T}\mathrm{PA}\right),\;\;\sigma_{x}=\sqrt{\text{diag}\left(Q_{\mathrm{xx}}\right)}$$ (6)

where mse is mean squared error, $Q_{\mathrm{xx}}$ is covariance matrix of unknowns, and *x* is the SDs of the estimates (i.e., $\sigma_{\text{ns}}$, $\sigma_{\text{ew}}$, and $\sigma_{\text{vlm}}$). Obtained decomposed North-South, East-West, and VLM rates with uncertainties are shown in fig. S8.

### InSAR-VLM validation

Our VLM estimates are validated against GNSS rates over 1994–2014 from UNR-NGL (*16*), NOAA-CORS [Continuously Operating Reference Station network; (*60*)], ESESES-MEaSUREs [Enhanced Solid Earth Science ESDR System; (*61*)], GAGE-PBO [Plate Boundary Observatory; (*62*)], and JPL-GeoGateway (*63*), all of which assume linear motion (fig. S9). Therefore, sites with nonlinear motion are excluded by applying a temporal variability threshold of 2.9 mm/year (90th percentile) calculated from the full-record NGL GNSS vertical time series. Next, we select GNSS sites that exist in all five solutions (*N* = 170) and compare these with the mean InSAR-VLM estimates (2015–2023) from within a 500-m radius around site. Our validation rationale is that InSAR-VLM captures the same long-term linear trends as those from the much longer GNSS record. We calculate the root mean square error (RMSE) between our InSAR-VLM estimates and each GNSS solution and use an ensemble mean to report the accuracy of our results (fig. S10), resulting in 1.8 mm/year. Among the different solutions, we found that our VLM agrees the best with the ESESES-MEaSUREs solution, with an RMSE of 1.6 mm/year, and shows the least agreement with GAGE-PBO, which has an RMSE of 2.0 mm/year. We note that some differences may arise in morphodynamically active areas (*9*), as InSAR observes land-surface response from both deep and shallow processes, while GNSS commonly captures only deep processes (depending on the anchoring depth of the GNSS benchmark) (*64*).

### Time-series LOS decomposition

We estimate the angle between calibrated and InSAR LOS linear trends, using the first epoch as a starting point. This angle is then used to rotate the uncalibrated LOS time series to match the trend direction of the calibrated one. After aligning all time series with the calibrated trends, we estimate an additive model with a constant trend, seasonality parameters (annual and semiannual), and automatic detection of change points for all time series, using the Prophet forecasting procedure (*65*). Once we obtain a best-fit model for all time-series locations, these models are used to interpolate LOS time series from different satellite tracks on the same temporal grid. This accounts for the differences in acquisition dates between satellite tracks from either ascending or descending orbits. The temporal grid is sampled every 12 days from 1 January 2016 to the end of the record. After calibrating and interpolating the time series for both ascending and descending orbit tracks to the same grid, we use the LOS decomposition equation from Eq. 5 to derive East-West and vertical time series. Because of the model’s adaptability to changing trends, we preserve nonlinear motion in the time series to a certain degree.

### Sea level projections with InSAR-VLM

We use relative sea level projections of the IPCC AR6 (*3*) on the basis of the Framework for Assessing Changes to Sea level (*7*). We focus on VLM contributions to projections in the near-term, by 2050, at point where SSP different scenarios (SSP2-4.5, SSP3-7.0, and SSP5-8.5) do not differ substantially (*2*, *3*, *44*). Therefore, we use the SSP2-4.5 scenario, a medium pathway of future emissions, with medium confidence contributions from ocean dynamics, glaciers, ice sheets, land water storage, and VLM at tide gauges. Here, we replace the framework’s VLM component of IPCC AR6 (*6*, *7*) with contemporary estimates of the GPS imaging (*10*), ALT-TG (*41*), and InSAR-VLM from this study, spatially averaged with data in radius of 500 m. We calculate the confidence interval for contemporary estimates using the *z*-score in a normal distribution to convert mean VLM rates and their uncertainties (SDs) to the 17th and 83rd percentiles. The outputs are relative sea level projections shown with ensemble median and confidence intervals defined by the 17th to 83rd percentiles at tide gauges. In the case of local VLM projections, we estimate projections uncertainties (17th to 83rd percentiles) using a probabilistic approach (*65*) with 2000 iterations of Markov chain Monte Carlo sampling.

## Supplementary Materials

### The PDF file includes:

Figs. S1 to S11 Tables S1 and S2, S4 to S7 Legend for table S3 References
