# Supplementary material for: Variable vertical land motion and its impacts on sea level rise projections
Source: Sci Adv. 2025 Jan 29;11(5):eads8163. doi: 10.1126/sciadv.ads8163 (PMC11777200; doi:10.1126/sciadv.ads8163)
Supplement: Supplementary file 1 — Figs. S1 to S11 Tables S1 and S2, S4 to S7 Legend for table S3 References [file sciadv.ads8163_sm.pdf]

Science Advances
AAAS

Supplementary Materials for

Variable vertical land motion and its impacts on sea level rise projections

Marin Govorcin *et al.*

Corresponding author: Marin Govorcin, marin.govorcin@jpl.nasa.gov

Sci. Adv. **11**, eads8163 (2025)
DOI: 10.1126/sciadv.ads8163

The PDF file includes:

Figs. S1 to S11
Tables S1 and S2, S4 to S7
Legend for table S3
References

Other Supplementary Material for this manuscript includes the following:

Table S3

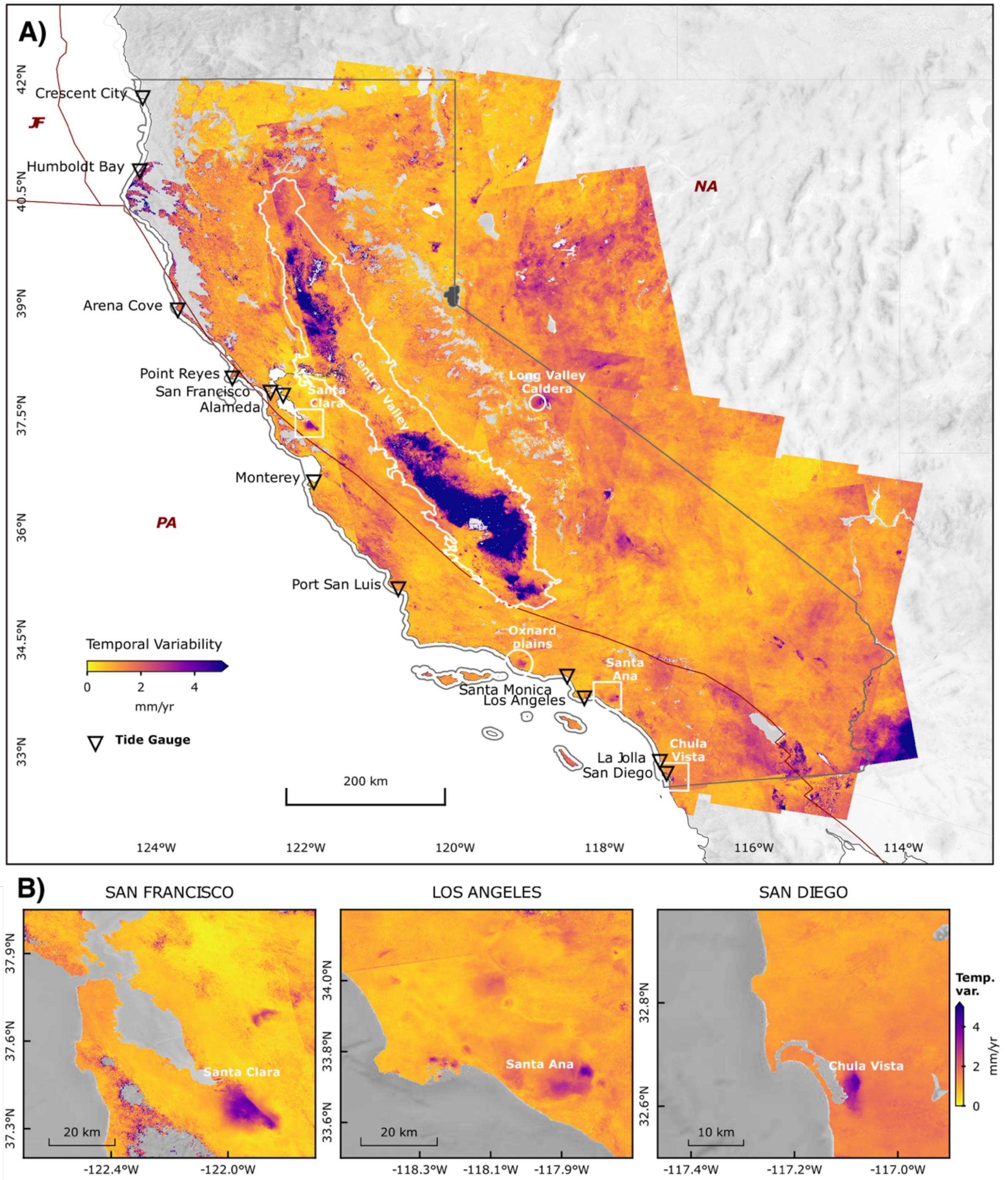

Fig S1. Temporal variability metric over California. (A) Temporal variability metric over the state of California for 2015-2023, showing the level of agreement between the trend estimated from the full record trend and the trends from different shorter segments (length > 3 years) of the record (see Methods). Higher values indicate a larger deviation from the linear trend. Here, temporal vertical land motion trend variations associated with the groundwater depletion and recharging are highlighted in (B), e.g. Central Valley, Santa Clara, Oxnard plains, Santa Ana, Chula Vista, as well as variations linked to episodic deflation-inflation events at Long Valley Caldera in A). Tectonic plate boundaries (maroon lines) follow: PA: Pacific plate, NA: North America plate, and JF: Juan de Fuca plate (**66**)

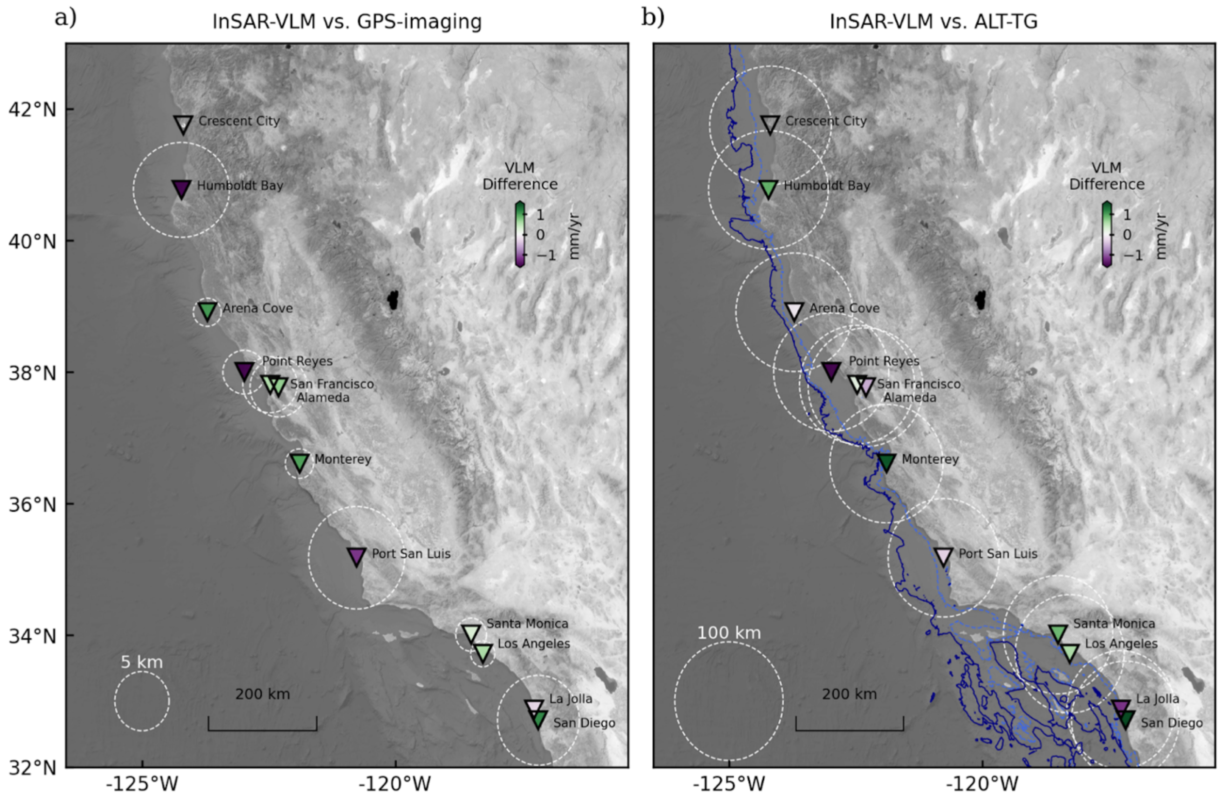

Fig. S2. Difference between contemporary VLM estimates at tide-gauges.
Comparison of contemporary VLM estimates at tide-gauges, (A) shows differences between InSAR-VLM and GPS-imaging estimates (**10**) with white circles (scaled by factor of 10) marking the distance from the closest contributing GNSS site to the GPS-imaging estimate. (B) shows the differences between InSAR-VLM and ALT-TG estimates (**41**), with white circles showing the extent of ALT-TG spatial resolution (1 degree = ~100km). Light and dark blue lines are 200- and 1000-meters isobaths derived from GEBCO2023 bathymetry data (available at https://www.gebco.net/).

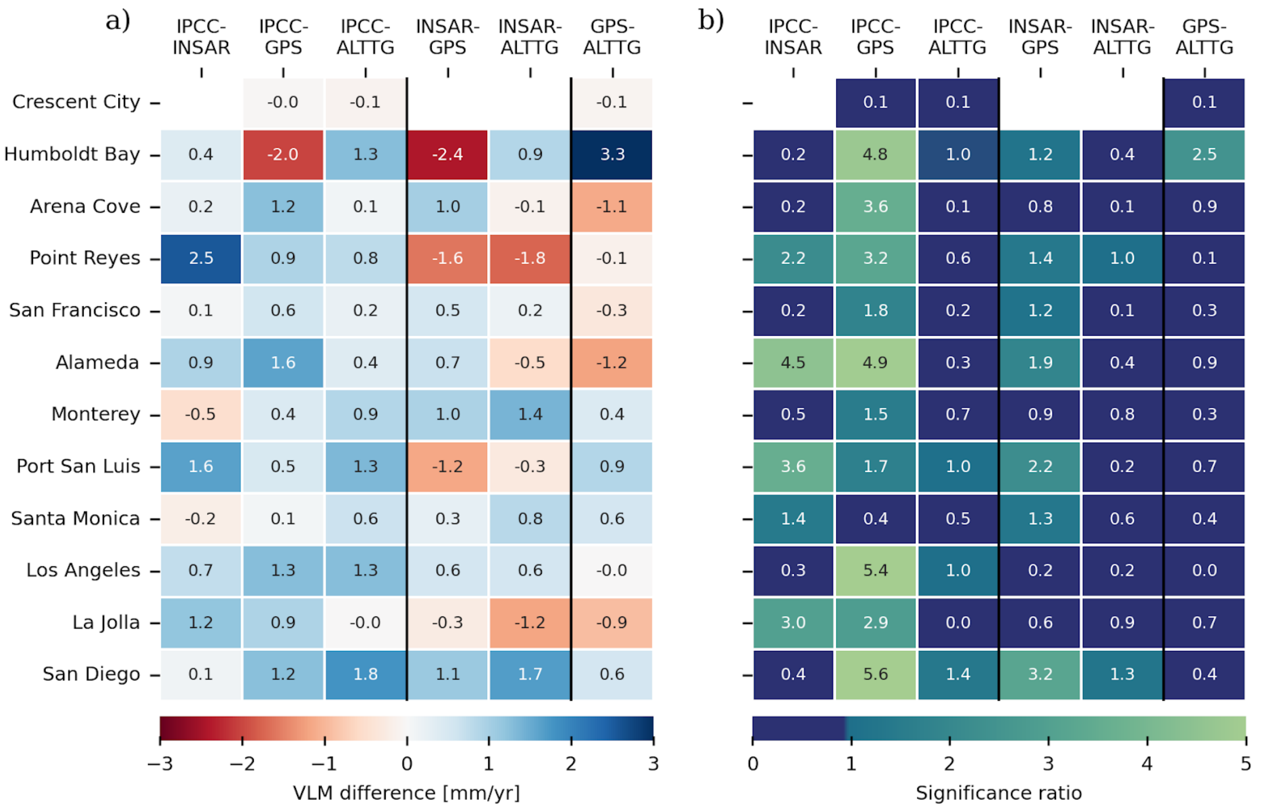

Fig S3. VLM differences at tide-gauges with associated significance ratio.
(A) comparison between different VLM estimates [IPCC-VLM; (**6**)], InSAR-VLM (this study), GPS-imaging (**10**), and ALT-TG (**41**) at tide-gauges, (B) significance ratio (**43**), for each value in a), calculated as $SR = \sqrt{(VLM_1 - VLM_1)^2/(\sigma_1^2 + \sigma_2^2)}$

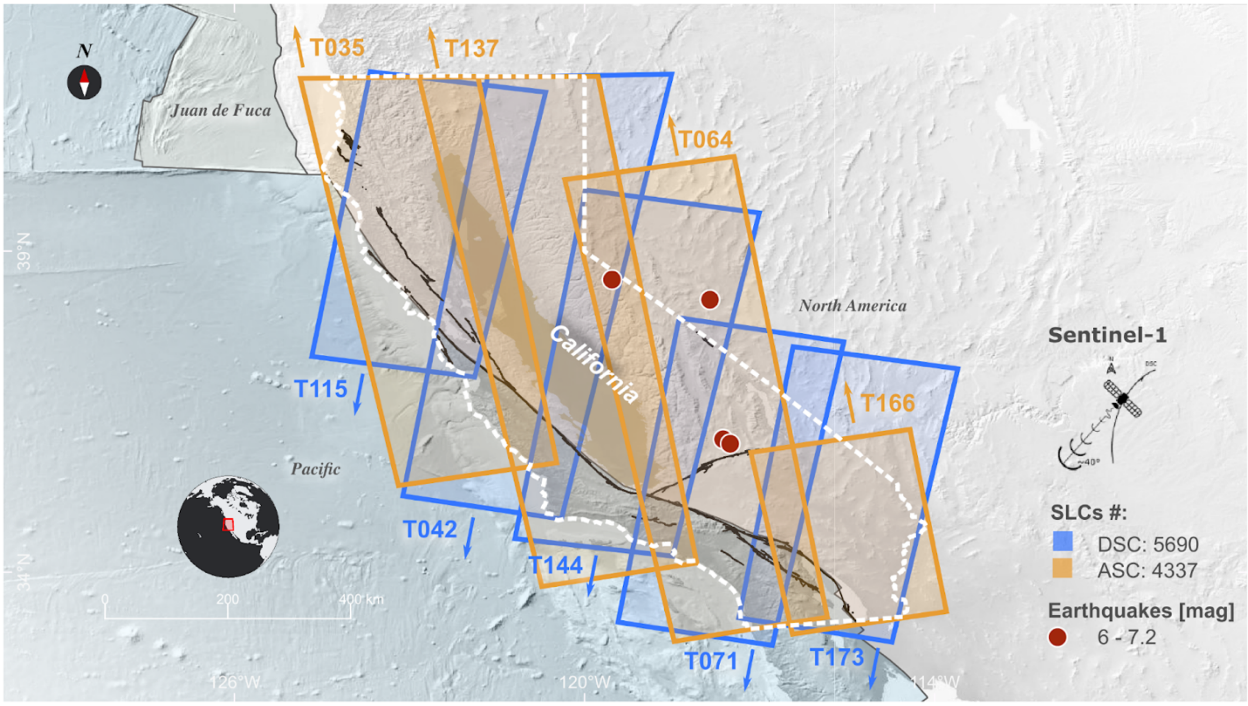

Fig. S4. Overview of used Sentinel-1 satellite tracks over California.

Overview of used Sentinel-1 tracks over the state of California, shown in orange (ascending orbits) and blue rectangles (descending orbits). Locations of significant earthquakes (U.S. Geological Survey, 2024, Earthquake List, https://www.usgs.gov/natural-hazards/earthquake-hazards, accessed June 05, 2024) during the observation period are marked with red circles (**Table S4**). Tectonic plates (**66**) are labeled and depicted in different colors, while the simplified San Andreas Fault (**70**) is illustrated with black lines.

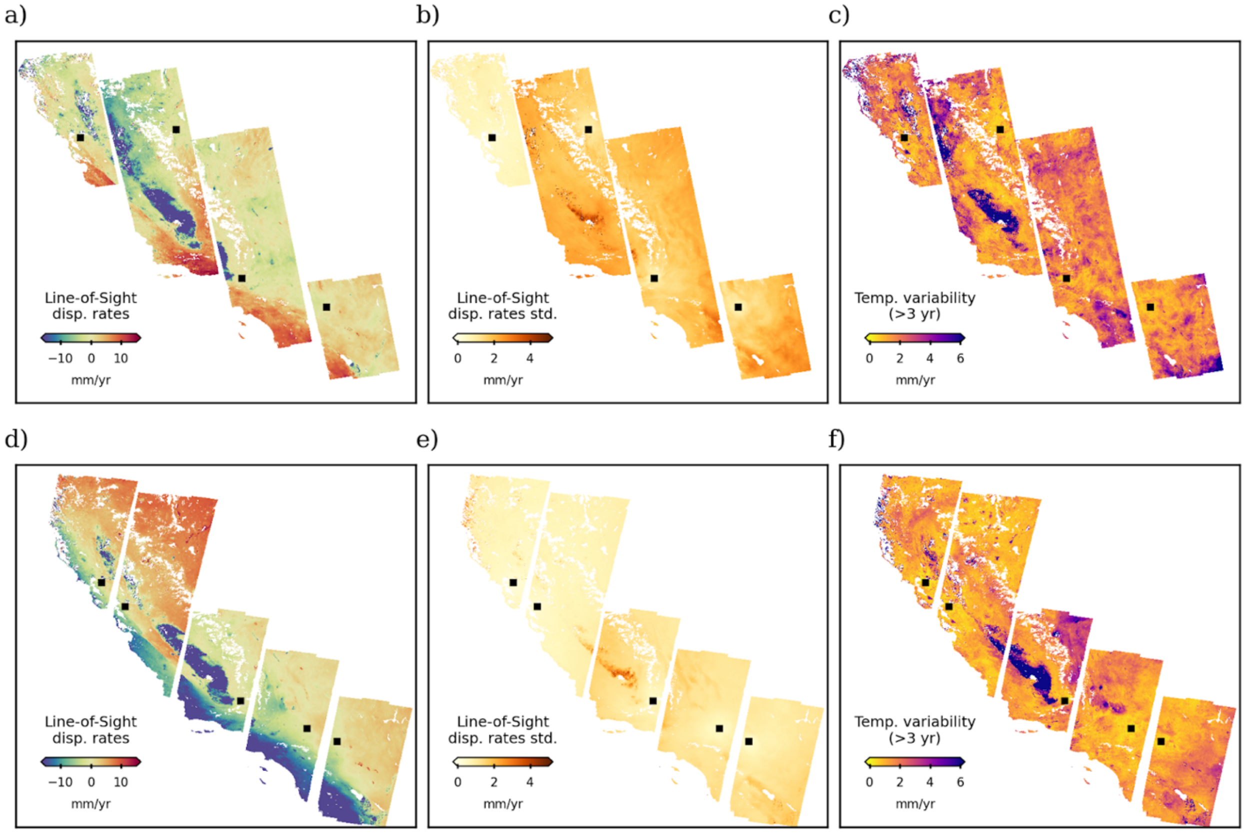

Fig. S5. Relative Sentinel-1 line-of-sight displacement rates with uncertainties.
Sentinel-1 relative line-of-sight displacement rates with associated formal uncertainties and temporal variability metric, where (A-C) show the latter for ascending orbit tracks, and (D-F) for descending orbit tracks. Displacements are relative to the arbitrary defined local reference point, marked as black squares. Positive values show motion towards and negative away from the satellite, along the satellite's imaging incidence angle.

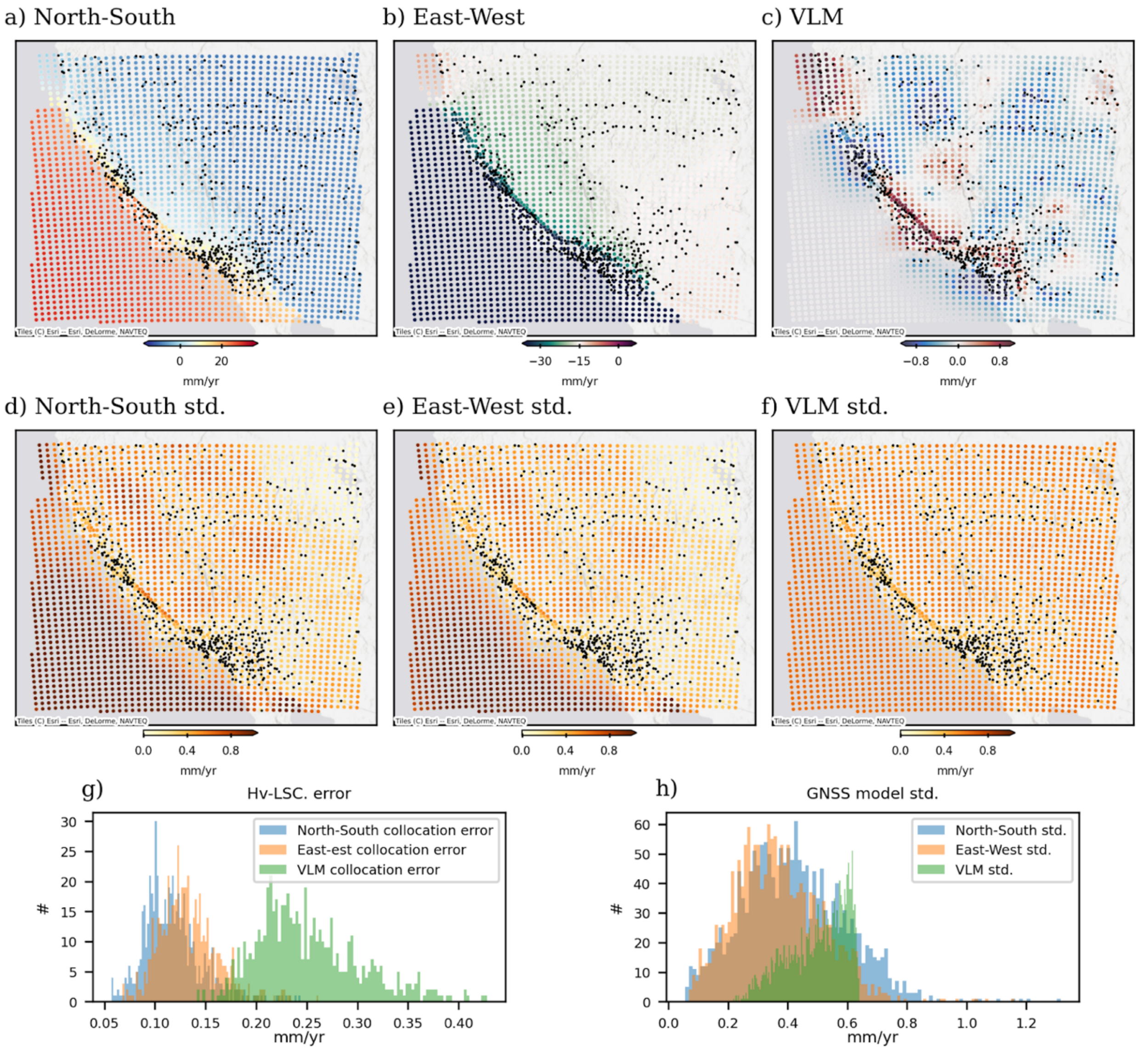

Fig. S6. GNSS model for California.

GNSS model on 25 km and 25 km grid for (A) North-South, (B) East-West, and (C) VLM long-term displacement rates, obtained with Hv-LSC-ex2 (**57**) approach from the preselected 'stable' GNSS sites (black dots). The grid is densified along the San Andreas Fault (**Fig. S4)**, to account for near-field fault motion. (D-F) are model grid uncertainties for each displacement component, combining both input measurement and model (covariance function) uncertainties (G) is Hv-LSC precision estimates, comparing the model with input values, indicating the vertical component as twice as uncertain as the horizontal components, consistent with the previous literature on GNSS precision (**71**), (H) show distribution of GNSS model uncertainties, excluding values over the ocean.

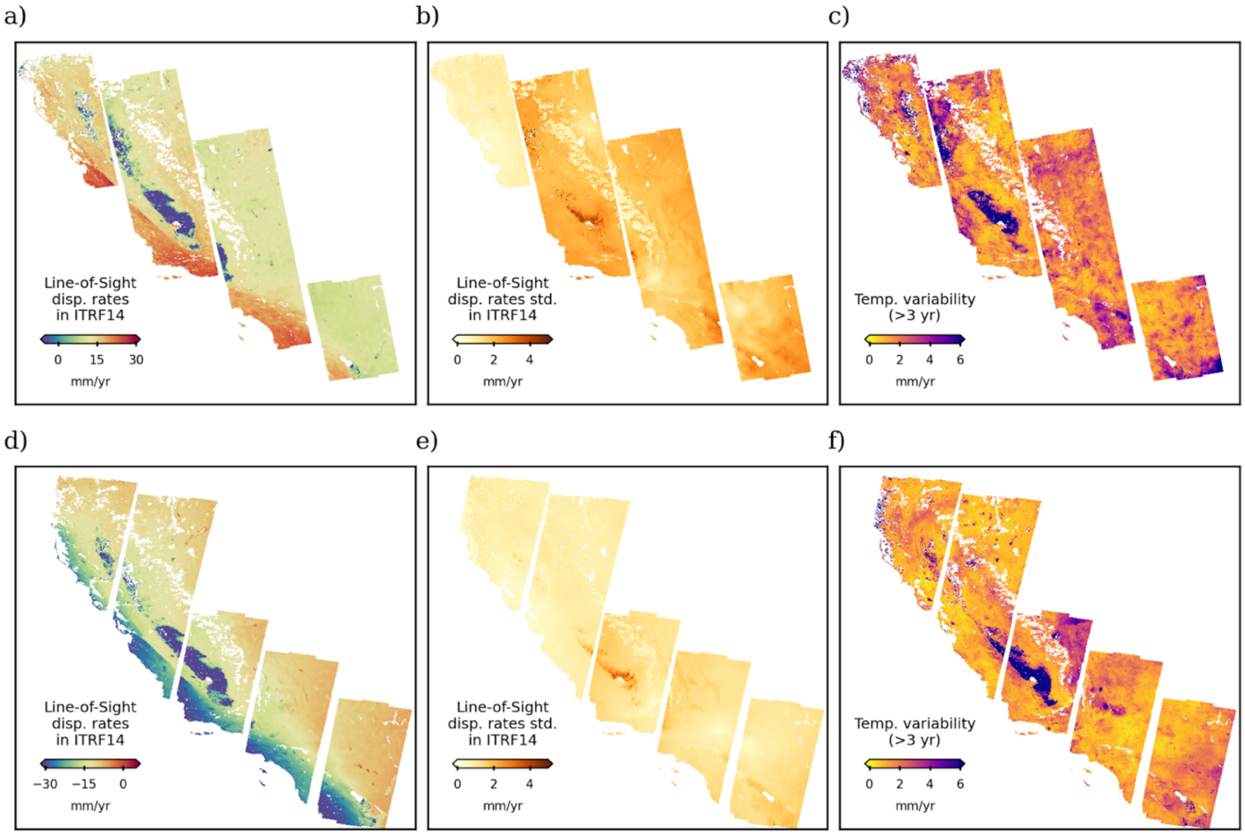

Fig. S7. Calibrated Sentinel-1 line-of-sight displacement rates in ITRF14.

(A) ascending tracks and (D) descending tracks showing LOS displacement rates, calibrated with the GNSS model from Figure S6. The calibration approach is detailed in the Methods section. (B) ascending and (E) descending tracks formal uncertainties for the calibrated rates, combining LOS uncertainties (**Fig S5**) and GNSS model uncertainties (**Fig. S6**). The displacement rates are relative to the ITRF2014 geodetic reference frame (**59**). Post-calibration, all tracks align and display the same long-wavelength signal, primarily associated with plate motion (plates are shown in **Fig. S4**). (C) ascending and (F) descending track temporal variability metric, same as in **Fig S5**.

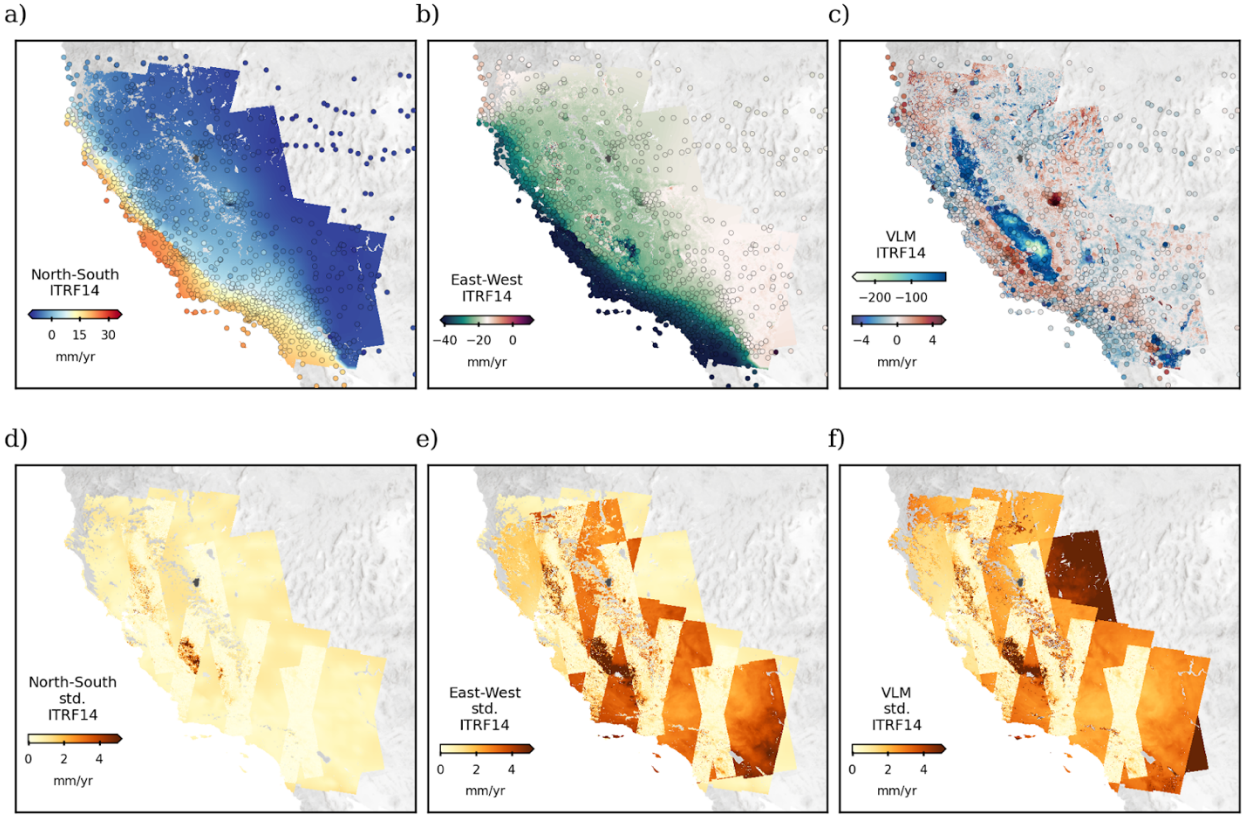

Fig. S8. North-South, East-West, and VLM land motion w.r.t ITRF14.

North-South, East-West, and VLM rates, with uncertainties, relative to the ITRF14 reference frame**,** estimated by line-of-sight (LOS) decomposition (outlined in the Methods section) of calibrated LOS displacement rates, with the addition of constraining north-south motion using a GNSS model sampled on the same grid. (A) North-South motion with (D) uncertainties mostly coming from the GNSS model. (B) East-West motion with (E) uncertainties, (C) VLM with (F) uncertainties. The circles show GNSS sites and displacement rates for 2012-2023, estimated from NGL time-series (**16**) with Hector software (**54**).

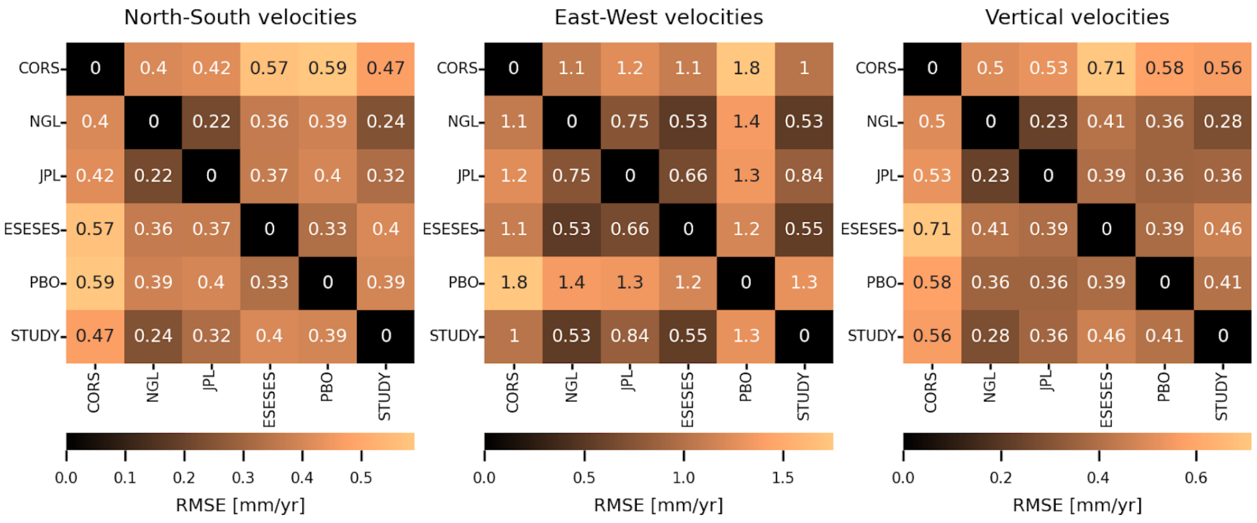

Fig. S9. GNSS velocity solutions from different providers.

Comparison of GNSS velocity solutions from different providers over California, for each displacement component: North-South, East-West, Vertical. The level of agreement between solutions is assessed using the root-mean-square error on a sample of 200 sites common to all solutions. The solutions compared include this study's estimates for 2012-2023, and other solutions estimated from the full GNSS records available from 1994-2024. Basic descriptions of each GNSS solution are provided in **Table S6**, while the ensemble mean is reported in **Table S7.** Similar findings on solution inconsistencies and possible source between different providers in previous studies (**72**).

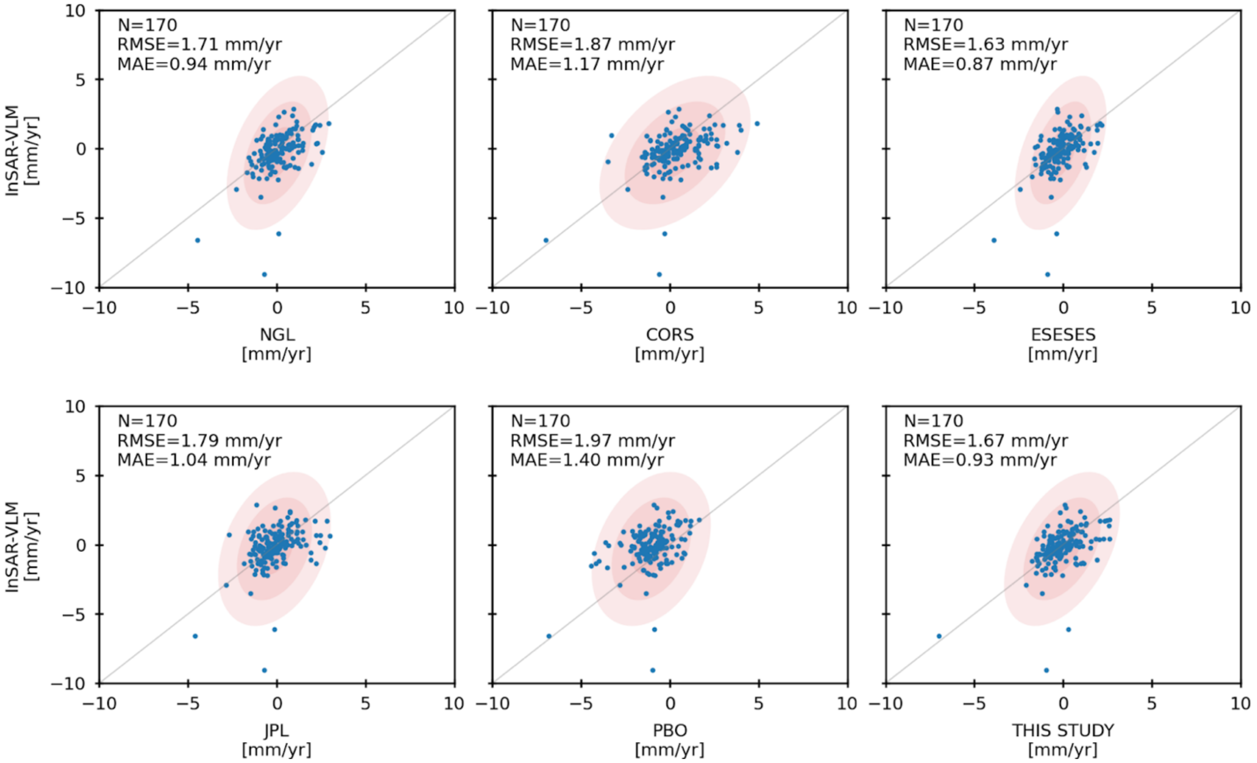

Fig. S10. InSAR-VLM Validation.

Validation of InSAR-derived Vertical Land Motion (VLM) is achieved by comparing the mean InSAR-VLM (2015-2023) within 500 meters around 170 GNSS sites that are common to five different GNSS solution (1994-2024) providers. A comparison between the GNSS solutions is provided in **Fig. S9**, and basic descriptions are detailed in **Table S6.** The level of agreement between InSAR-VLM and GNSS solutions is quantified using root mean square error (RMSE) and mean absolute error (MAE).

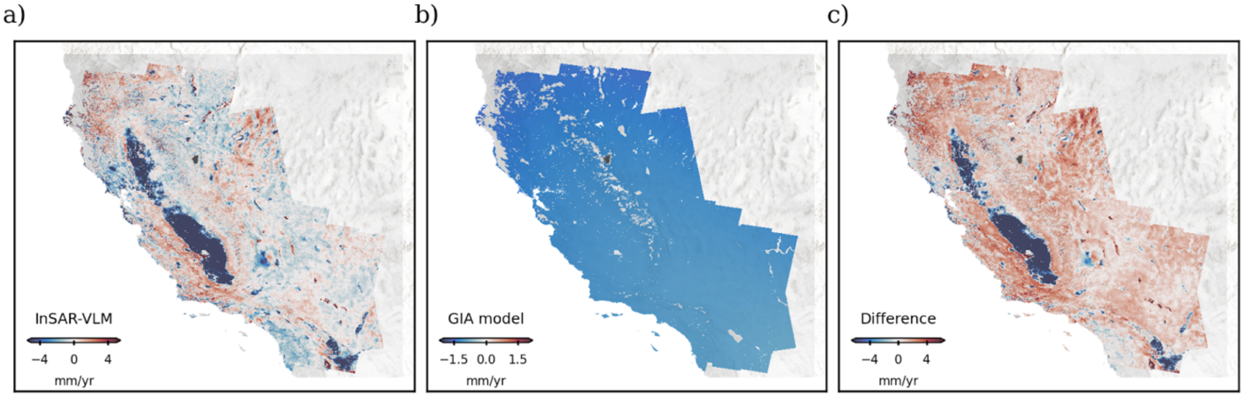

Fig. S11. Glacial Isostatic Adjustment (GIA) model and InSAR-VLM.

Comparison with glacial isostatic adjustment (GIA) model estimates**,** (A) InSAR-VLM, (B) GIA estimates (**73**) sample on the InSAR-VLM grid, (C) differences between INSAR-VLM and the GIA estimates.

Table S1. Local Vertical Land Motion (VLM) projections by 2050.

Vertical Land Motion (VLM) projections in 2050 at selected spots from **Fig 4**, with respect to reference date January 01, 2016. VLM displacements are expressed in ITRF14 frame.

Location	Selected Spot	Median [meters]	17th [meters]	83rd [meters]	Uncertainty range [meters]
San Francisco Bay	San Rafael	-0.32	-0.38	-0.25	0.13
	Corte Madera	-0.28	-0.34	-0.23	0.11
	West Contra Costa Sanitary Landfill	-0.27	-0.35	-0.19	0.17
	San Francisco Airport	-0.14	-0.19	-0.08	0.11
	Bay Farm City	-0.16	-0.21	-0.11	0.10
	Foster City	-0.09	-0.13	-0.05	0.08
	San Francisco Tide Gauge	-0.03	-0.06	-0.01	0.06
	Alameda Tide Gauge	-0.02	-0.04	0.01	0.05
Los Angeles	Palos Verdes	-0.24	-0.29	-0.18	0.11
	Belmont Shores	-0.04	-0.06	-0.02	0.04
	Newport Beach	-0.10	-0.12	-0.07	0.05
	Long Beach Shoreline Marina	-0.14	-0.18	-0.11	0.07
	Long Beach – injection	0.23	0.11	0.36	0.25
	Long Beach - extraction	0.09	-0.12	0.30	0.41
	Santa Monica Tide Gauge	0.02	-0.00	0.03	0.04
	Los Angeles Tide Gauge	-0.00	-0.02	0.01	0.04
San Diego	Torrey Pines- Black beach	-0.10	-0.12	-0.07	0.06
	Mission Bay	-0.05	-0.08	-0.01	0.07
	San Diego Airport	-0.02	-0.05	0.01	0.07
	Chula Vista	-0.04	-0.20	0.14	0.34
	San Diego Tide Gauge	-0.01	-0.04	0.01	0.06
	La Jolla Tide Gauge	-0.04	-0.06	-0.00	0.06

Table S2. Overview of used ARIA S1-GUNW products.
Overview of the Advanced Rapid and Imaging Analysis (ARIA) Sentinel-1 Geocoded Unwrapped Phase (S1-GUNW) products over California, with satellite orbit track number, orbit direction, number of products, and date range and number of satellite acquisitions. Number of ARIA-S1-GUNW products count more than acquisition dates, as we use multiple products along the same track that are stitched with ARIA-tools (**12**), with the new sequential stitcher described in Methods.

**Sentinel-1 Track Number**	**Orbit Directions**	**Start Date**	**End Date**	**Number of ARIA-GUNW products**	**Number of SAR acquisition dates**
35	Ascending	2015-05-24	2022-06-16	6951	806
64	Ascending	2014-11-27	2022-06-06	8336	1159
137	Ascending	2015-05-24	2023-08-29	15017	1676
166	Ascending	2015-04-03	2021-12-21	3309	696
42	Descending	2015-03-01	2022-06-04	6080	938
71	Descending	2015-05-14	2022-06-06	5432	1122
115	Descending	2015-02-22	2022-05-16	4177	892
144	Descending	2015-03-01	2023-08-29	9433	2090
173	Descending	2014-12-04	2022-06-01	2756	648

Table S3. List of added and removed steps in timeseries model fitting.

List of added and removed steps (from the UNR step archive; http://geodesy.unr.edu/NGLStationPages/steps.txt for some GNSS sites, after visual inspection) (provided as external file)

Table S4. Statistics for each track.

Statistics (median and interquartile range) for line-of-sight displacement rates, formal uncertainties (standard deviation) and temporal variability metric for each track, with acquisition local time and number of used Heaviside steps (for earthquake magnitude >6 within the observations period: M7.1 Ridgecrest - 20190705, 20190706, M6.5 Monte Cristo - 20200515, M6 Antelope Valley - 20210708). Here, we observe 1) ascending orbit tracks exhibit higher uncertainties compared to descending tracks, likely due to increased turbulent atmosphere in the afternoon, 2) adding Heaviside steps tends to increase formal uncertainties by a factor or two, possibly due to overfitting in the areas distant from the earthquake epicenter, as the same functional fitting parameters are applied across the track. Track's satellite orbit direction (ascending or descending) is in **Table-S1**.

**Sentinel-1 Track Number**	**Median LOS std [mm/yr]**	**IQR LOS std [mm/yr]**	**Median LOS temp.var. [mm/yr]**	**IQR LOS temp.var. [mm/yr]**	**Local time (Pacific Standard Time) of acquisitions [HH:MM:ss]**	**Heaviside steps**
35	0.68	0.14	1.79	1.4	18:07:44	**-**
64	1.88	0.37	1.89	0.98	17:50:02	**20190705, 20200515**
137	2.01	0.43	1.52	1.47	17:59:25	**20190705, 20210708**
166	1.94	0.75	1.65	1.09	17:41:47	**20190706**
42	0.71	0.11	1.04	0.74	06:07:31	**-**
71	1.15	0.25	1.26	0.83	05:51:50	**20190705**
115	0.66	0.16	1.24	0.87	06:15;24	**-**
144	1.21	0.37	1.84	1.81	05:59:53	**20190706, 20200515**
173	1.15	0.34	1.50	0.84	05:43:54	**20190705**

Table S5. VLM with formal uncertainties at tide-gauges.

VLM estimates with formal uncertainties at tide-gauges along the California coast. InSAR-VLM is estimated in this study as described in Methods, whereas other: IPCC-VLM and uncertainties (**6**,**7**) are from (**69**), GPS-imaging (**10**) estimates and uncertainties are downloaded from (http://geodesy.unr.edu/vlm.php), and ALT-TG estimates are from Hamlington et al (**41**), whereas, due to the missing uncertainties in (**41**), we use root-mean-square-error from (**40**) that used the same approach.

Tide gauge location	InSAR VLM rates [mm/yr]	InSAR VLM rates unc. 1σ [mm/yr]	GPS Imaging rates [mm/yr]	GPS Imaging rates unc. 1σ [mm/yr]	IPCC VLM rates mm/yr	IPCC VLM rates unc. 1σ mm/yr	ALT-TG VLM rates [mm/yr]	ALT-TG VLM rates unc. 1σ mm/yr
Crescent City	-	-	2.4	0.3	2.4	0.1	2.5	1.3
Humboldt Bay	-2.9	2.0	-0.5	0.3	-2.5	0.3	-3.8	1.3
Arena Cove	0.4	1.2	-0.6	0.2	0.6	0.3	0.5	1.3
Point Reyes	-2.8	1.1	-1.2	0.2	-0.2	0.2	-1.0	1.3
San Francisco	-0.3	0.3	-0.8	0.3	-0.2	0.1	-0.5	1.3
Alameda	-0.2	0.2	-0.9	0.3	0.7	0.1	0.3	1.3
Monterey	0.8	1.1	-0.2	0.2	0.3	0.2	-0.6	1.3
Port San Luis	-0.8	0.4	0.3	0.3	0.8	0.1	-0.6	1.3
Santa Monica	0.4	0.1	0.2	0.2	0.2	0.1	-0.4	1.3
Los Angeles	0.0	2.3	-0.6	0.2	0.7	0.1	-0.6	1.3
La Jolla	-1.5	0.3	-1.3	0.3	-0.3	0.2	-0.3	1.3
San Diego	-0.6	0.3	-1.8	0.2	-0.5	0.1	-2.3	1.3

Table S6. Brief description on GNSS solutions from different providers.

Brief description on GNSS solutions from different providers, including their time series and velocities analysis approach: CORS: National Oceanic and Atmospheric Administration's Continuously operating Reference Stations (CORS) network available at (https://geodesy.noaa.gov/CORS/data.shtml), NGL: University of Nevada, Reno, Nevada Geodetic Laboratory (NGL) available at (http://geodesy.unr.edu/), JPL: GeoGateway project at the Jet Propulsion Laboratory (JPL) available at (https://sideshow.jpl.nasa.gov/post/tables/table2.html), ESESES: Extended Solid Earth Science ESDR System (ESESES) project funded by NASA MEASUREs available at (https://cddis.nasa.gov/Data_and_Derived_Products/GNSS/MEaSUREs/), PBO: Plate Boundary Observatory (PBO) from NSF GAGE-Earthscope program available at (https://www.unavco.org/data/gps-gnss/derived-products/derived-products.html). The difference in processing approaches could be due to 1) daily positions processing with double-differencing (GAMIT: (**74**) or precise point position [PPP, GipsyX : (**75**)] 2) trend and its uncertainty estimation either using parametric (functional fitting) or nonparametric [MIDAS: (**76**)], with different stochastic models for uncertainties (**77**) , 3) tackling abrupt changes (steps) in GNSS times series: using visual inspection, automatic detection (**63**), or archive of reported instrument changes and nearby earthquake events (e.g. UNR-NGL step archive: http://geodesy.unr.edu/NGLStationPages/steps.txt)

GNSS provider	NOAA CORS	UNR NGL	ESESES MEaSUREs	JPL Geo Gateway	GAGE PBO	This study (UNR-NGL timeseries)
Record length	1994-01-01 present	1994-01-01 present	1992-06-13 present	1994-01-01 present	1996-01-01 2024-01-23	2012-01-01 2023-01-01
Ref. epoch	2010-01-01	2010-01-01	2010-01-01	2025-01-01	2023-12-27	2010-01-01
Ref. frame	IGS14	IGS14	IGS14	IGS14	IGS14	IGS14
Time series app.	**???**	GipsyX	Combined GipsyX and GAMIT	GipsyX	GAMIT	UNR-NGL time-series (GipsyX)
Model for linear rates	parametric fitting	Non Parametric MIDAS	parametric fitting	parametric fitting	parametric fitting	parametric fitting
Model unc.	modeled as white noise	scaled standard error in the median	modeled as colored noise	modeled as white noise	modeled as random walk noise	modeled as colored noise
Steps:	Visual inspection	Archive of reported instrument changes and earthquakes	Visual inspection	Automatic detection	Visual inspection	UNR-NGL Archive With Visual inspection

Table S7. Ensemble RMSE between different GNSS solutions.

Mean Root-Mean-Square Error Between Different GNSS Solutions. The mean root-mean-square error (RMSE) between different GNSS solutions (as detailed in **Table S5**) is calculated for each solution presented in **Fig. S6**. The ensemble mean RMSE is reported for each displacement component: North-South, East-West, and Vertical Land Motion (VLM).

N = 200 sites	East-West [mm/yr]	North-South [mm/yr]	VLM [mm/yr]
NOAA-CORS	0.4	0.5	1.0
UNR-NGL	0.3	0.3	0.7
ESESES-MEaSUREs	0.3	0.4	0.7
JPL-GeoGateway	0.3	0.3	0.8
GAGE-PBO	0.3	0.3	1.1
This study (UNR-NGL time series fitted with Hector)	0.3	0.3	0.7
ENSEMBLE MEAN	0.3	0.4	0.8

REFERENCES AND NOTES

1. J. M. Gregory, S. M. Griffies, C. W. Hughes, J. A. Lowe, J. A. Church, I. Fukimori, N. Gomez, R. E. Kopp, F. Landerer, G. L. Cozannet, R. M. Ponte, Concepts and terminology for sea level: Mean, variability and change, both local and global. *Surv. Geophysics* **40**, 1251–1289 (2019).

2. M. Oppenheimer, B. C. Glavovic, J. Hinkel, R. Wal, A. K. Magnan, A. Abd-Elgawad, R. Cai, M. Cifuentes-Jara, R. M. DeConto, T. Ghosh, J. Hay, F. Isla, B. Marzeion, B. Meyssignac, Z. Sebesvari, "Sea level rise and implications for low-lying islands, coasts and communities" in *IPCC Special Report on the Ocean and Cryosphere in a Changing Climate*, H.-O. Pörtner, D. C. Roberts, V. Masson-Delmotte, P. Zhai, M. Tignor, E. Poloczanska, K. Mintenbeck, A. Alegría, M. Nicolai, A. Okem, J. Petzold, B. Rama, N. M. Weyer, Eds. (Cambridge Univ. Press, 2019), pp. 321–445.

3. B. Fox-Kemper, H. T. Hewitt, C. Xiao, G. Adalgeirsdottir, S. S. Drijfhout, T. L. Edwards, N. R. Golledge, M. Hemer, R. E. Kopp, G. Krinner, A. Mix, D. Notz, S. Nowicki, I. S. Nurhati, L. Ruiz, J.-B. Sallée, A. B. A. Slangen, Y. Yu, "Ocean, cryosphere and sea level change" in *Climate Change 2021: The Physical Science Basis. Contribution of Working Group I to the Sixth Assessment Report of the Intergovernmental Panel on Climate Change*, V. Masson-Delmotte P. Zhai, A. Pirani, S. L. Connors, C. Péan, S. Berger, N. Caud, Y. Chen, L. Goldfarb, M. I. Gomis, M. Huang, K. Leitzell, E. Lonnoy, J. B. R. Matthews, T. K. Maycock, T. Waterfield, O. Yelekçi, R. Yu, B. Zhou, Eds. (Cambridge Univ. Press, 2021), pp. 1211–1362.

4. B. D. Hamlington, A. S. Gardner, E. Ivins, J. T. M. Lenaerts, J. T. Reager, D. S. Trossman, E. D. Zaron, S. Adhikari, A. Arendt, A. Aschwanden, B. D. Beckley, D. P. S. Bekaert, G. Blewitt, L. Caron, D. P. Chambers, H. A. Chandanpurkar, K. Christianson, B. Csatho, R. I. Cullather, R. M. DeConto, J. T. Fasullo, T. Frederikse, J. T. Freymueller, D. M. Gilford, M. Girotto, W. C. Hammond, R. Hock, N. Holschuh, R. E. Kopp, F. Landerer, E. Larour, D. Menemenlis, M. Merrifield, J. X. Mitrovica, R. S. Nerem, I. J. Nias, V. Nieves, S. Nowicki, K. Pangaluru, C. G. Piecuch, R. D. Ray, D. R. Rounce, N.-J. Schlegel, H. Seroussi, W. V. Sweet, I. Velicogna, N. Vinogradova, T. Wahl, D. N. Wiese, M. J. Willis, Understanding of contemporary regional sea-level change and the implications for the future. *Rev. Geophysics* **58**, e2019RG000672 (2020).

5. A. B. A. Slangen, M. D. Palmer, C. M. L. Camargo, J. A. Church, T. L. Edwards, T. H. J. Hermans, H. T. Hewitt, G. G. Garner, J. M. Gregory, R. E. Kopp, V. M. Santos, R. S. W. van de Wal, The evolution of 21st-century sea-level projections from IPCC AR5 to AR6 and beyond. *Camb. Prisms Coast. Futures* **1**, 7 (2023).

6. R. E. Kopp, R. M. Horton, C. M. Little, J. X. Mitrovica, M. Oppenheimer, D. J. Rasmussen, B. H. Strauss, C. Tebaldi, Probabilistic 21st and 22nd-century sea-level projections at a global network of tide-gauge sites. *Earth's Future* **2**, 383–406 (2014).

7. R. E. Kopp, G. G. Garner, T. H. J. Hermans, S. Jha, P. Kumar, A. Reedy, A. B. A. Slangen, M. Turilli, T. L. Edwards, J. M. Gregory, G. Koubbe, A. Levermann, S. Merzky, S. Nowicki, M. D. Palmer, C. Smith, The framework for assessing changes to sea-level (FACTS) v1.0: A platform for characterizing parametric and structural uncertainty in future global, relative, and extreme sea-level change. *Geosci. Model Dev.* **16**, 7461–7489 (2023).

8. M. Shirzaei, J. Freymueller, T. E. Törnqvist, D. L. Galloway, T. Dura, P. S. J. Minderhoud, Measuring, modelling and projecting coastal land subsidence. *Nat Rev Earth Environ.* **2**, 40–58 (2021).

9. T. E. Törnqvist, M. D. Blum, What is coastal subsidence? *Camb. Prisms Coast. Futures* **2**, e2 (2024).

10. W. C. Hammond, G. Blewitt, C. Kreemer, R. S. Nerem, GPS imaging of global vertical land motion for studies of sea-level rise. *J. Geophys. Res. Solid Earth* **126**, e2021JB022355 (2021).

11. M. Shirzaei, R. Bürgmann, Global climate change and local land subsidence exacerbate inundation risk to the San Francisco Bay Area. *Sci. Adv.* **4**, eaap9234 (2018).

12. B. Buzzanga, D. P. S. Bekaert, B. D. Hamlington, S. S. Sangha, Toward sustained monitoring of subsidence at the coast using InSAR and GPS: An application in Hampton Roads, Virginia. *Geophy. Res. Lett.* **47**, e2020GL090013 (2020).

13. B. Buzzanga, D. P. S. Bekaert, B. D. Hamlington, R. E. Kopp, M. Govorcin, K. G. Miller, Localized uplift, widespread subsidence, and implications for sea-level rise in the New York City metropolitan area. *Sci. Adv.* **9**, eade8259 (2023).

14. L. O. Ohenhen, M. Shirzaei, C. Ojha, S. F. Sherpa, R. J. Nicholls, Disappearing cities on US coasts. *Nature* **627**, 108–115 (2024).

15. D. P. S. Bekaert, C. E. Jones, K. An, M.-H. Huang, Exploiting UAVSAR for a comprehensive analysis of subsidence in the Sacramento Delta. *Remote Sens. Environ.* **220**, 124–134 (2019).

16. G. Blewitt, W. C. Hammond, C. Kreemer, Harnessing the GPS data explosion for interdisciplinary science. *Eos* **99**, 10.1029/2018EO104623 (2018).

17. G. Li, C. Zhao, B. Wang, M. Peng, L. Bai, Evolution of spatiotemporal ground deformation over 30 years in Xi'an, China, with multi-sensor SAR interferometry. *J. Hydrol.* **616**, 128764 (2023).

18. E. Blackwell, M. Shirzaei, C. Ojha, S. Werth, Tracking California's sinking coast from space: Implications for relative sea-level rise. *Sci. Adv.* **6**, eaba4551 (2020).

19. C. Tay, E. O. Lindsey, S. T. Chin, J. W. McCaughey, D. Bekaert, M. Nguyen, H. Hua, G. Manipon, M. Karim, B. P. Horton, T. Li, E. M. Hill, Sea-level rise from land subsidence in major coastal cities. *Nat. Sustainability* **5**, 1049–1057 (2022).

20. Z. Ao, X. Hu, S. Tao, X. Hu, G. Wang, M. Li, F. Wang, L. Hu, X. Liang, J. Xiao, A national-scale assessment of land subsidence in China's major cities. *Science* **384**, 301–306 (2024).

21. T. Naish, R. Levy, I. Hamling, S. Hreinsdóttir, P. Kumar, G. G. Garner, R. E. Kopp, N. Golledge, R. Bell, R. Paulik, J. Lawrence, P. Denys, T. Gillies, S. Bengtson, A. Howell, K. Clark, D. King, N. Litchfield, R. Newnham, The significance of interseismic vertical land movement at convergent plate boundaries in probabilistic sea-level projections for AR6 scenarios: The New Zealand case. *Earth's Future* **12**, e2023EF004165 (2024).

22. J. Oelsmann, M. Marcos, M. Passaro, L. Sanchez, D. Dettmering, S. Dangendorf, F. Seitz, Regional variations in relative sea-level changes influenced by nonlinear vertical land motion. *Nat. Geosci.* **17**, 137–144 (2024).

23. W. C. Hammond, G. Blewitt, C. Kreemer, GPS imaging of vertical land motion in California and Nevada: Implications for Sierra Nevada uplift. *J. Geophys. Res. Solid Earth* **121**, 7681–7703 (2016).

24. E. Klein, Y. Bock, X. Xu, D. T. Sandwell, D. Golriz, P. Fang, L. Su, Transient deformation in California from two decades of GPS displacements: Implications for a three-dimensional kinematic reference frame. *J. Geophys. Res. Solid Earth* **124**, 12189–12223 (2019).

25. B. R. Smith-Konter, G. M. Thornton, D. T. Sandwell, Vertical crustal displacement due to interseismic deformation along the San Andreas fault: Constraints from tide gauges. *Geophys. Res. Lett.* **41**, 3793–3801 (2014).

26. L. A. Ward, K. A. Guns, B. R. Smith-Konter, X. Xu, Y. Bock, D. T. Sandwell, Vertical postseismic deformation of the 2019 Ridgecrest earthquake sequence. *J. Geophys. Res. Solid Earth* **127**, e2021JB023331 (2022).

27. F. Silverii, F. Pulvirenti, E. K. Montgomery-Brown, A. A. Borsa, W. R. Neely, The 2011-2019 Long Valley Caldera inflation: New insights from separation of superimposed geodetic signals and 3D modeling. *Earth Planet. Sci. Lett.* **569**, 117055 (2021).

28. S. Jasechko, H. Seybold, D. Perrone, Y. Fan, M. Shamsudduha, R. G. Taylor, O. Fallatah, J. W. Kirchner, Rapid groundwater decline and some cases of recovery in aquifers globally. *Nature* **625**, 715–721 (2024).

29. A. L. Handwerger, E. J. Fielding, S. S. Sangha, D. P. S. Bekaert, Landslide sensitivity and response to precipitation changes in wet and dry climates. *Geophys. Res. Lett.* **49**, e2022GL099499 (2022).

30. Z. M. Swirad, A. P. Young, Spatial and temporal trends in California coastal cliff retreat. *Geomorphology* **412**, 108318 (2022).

31. G. Beach, Oceano dunes in the Guadalupe-Nipomo dunes complex. *Calif. Coast. Comm.* (2021).

32. X. Xu, D. T. Sandwell, E. Klein, Y. Bock, Integrated Sentinel-1 InSAR and GNSS time-series along the San Andreas fault system. *J. Geophys. Res. Solid Earth* **126**, e2021JB022579 (2021).

33. W. C. Hammond, R. J. Burgette, K. M. Johnson, G. Blewitt, Uplift of the western transverse ranges and Ventura area of Southern California: A four-technique geodetic study combining GPS, InSAR, leveling, and tide gauges. *J. Geophys. Res. Solid Earth* **123**, 836–858 (2018).

34. California Department of Water Resources (DWR): The Water Data Library (2023); https://wdl.water.ca.gov/WaterDataLibrary/.

35. M. Marvin-DiPasquale, D. Slotton, J. T. Ackerman, M. Downing-Kunz, B. E. Jaffe, A. C. Foxgrover, F. Achete, M. Wegen, "South San Francisco Bay Salt Pond Restoration Project—A synthesis of phase-1 mercury studies" (Tech. Rep. 2022-5113, US Geological Survey, 2022); https://doi.org/10.3133/sir20225113.

36. K. M. Befus, P. L. Barnard, D. J. Hoover, J. A. Finzi Hart, C. I. Voss, Increasing threat of coastal groundwater hazards from sea-level rise in California. *Nat. Clim. Change* **10**, 946–952 (2020).

37. National Research Council, *Sea-Level Rise for the Coasts of California, Oregon, and Washington: Past, Present, and Future* (The National Academies Press, 2012); https://doi.org/10.17226/13389.

38. P. L. Barnard, L. H. Erikson, A. C. Foxgrover, J. A. F. Hart, P. Limber, A. C. O'Neill, M. Ormondt, S. Vitousek, N. Wood, M. K. Hayden, J. M. Jones, Dynamic flood modeling essential to assess the coastal impacts of climate change. *Sci. Rep.* **9**, 4309 (2019).

39. J. C. Lee, M. Shirzaei, Novel algorithms for pair and pixel selection and atmospheric error correction in multitemporal InSAR. *Remote Sens. Environ.* **286**, 113447 (2023).

40. M. Kleinherenbrink, R. Riva, T. Frederikse, A comparison of methods to estimate vertical land motion trends from GNSS and altimetry at tide gauge stations. *Ocean Sci.* **14**, 187–204 (2018).

41. B. D. Hamlington, D. P. Chambers, T. Frederikse, S. Dangendorf, S. Fournier, B. Buzzanga, R. S. Nerem, Observation-based trajectory of future sea level for the coastal United States tracks near high-end model projections. *Commun. Earth Environ.* **3**, 230 (2022).

42. P. L. Woodworth, A. Melet, M. Marcos, R. D. Ray, G. Wöppelmann, Y. N. Sasaki, M. Cirano, A. Hibbert, J. M. Huthnance, S. Monserrat, M. A. Merrifield, Forcing factors affecting sea level changes at the coast. *Surv Geophys.* **40**, 1351–1397 (2019).

43. J. Pfeffer, G. Spada, A. Memin, J.-P. Boy, P. Allemand, Decoding the origins of vertical land motions observed today at coasts. *Geophys. J. Int.* **210**, 148–165 (2017).

44. W. V. Sweet, B. D. Hamlington, R. E. Kopp, C. P. Weaver, P. L. Barnard, D. Bekaert, W. Brooks, M. Craghan, G. Dusek, T. Frederikse, G. Garner, A. S. Genz, J. P. Krasting, E. Larour, D. Marcy, J. J. Marra, J. Obeysekera, M. Osler, M. Pendleton, D. Roman, L. Schmied, W. Veatch, K. D. White, C. Zuzak, "Global and regional sea level rise scenarios for the United States: Updated mean projections and extreme water level probabilities Along U.S. coastlines" (Tech. Rep. NOS 01, National Oceanic and Atmospheric Administration National Ocean Service, 2022); https://sealevel.globalchange.gov/resources/2022-sea-level-rise-technical-report/.

45. State of California, "Sea level rise guidance: 2024 Science and policy update" (California Ocean Protection Council, 2024).

46. R. J. Nicholls, D. Lincke, J. Hinkel, S. Brown, A. T. Vafeidis, B. Meyssignac, S. E. Hanson, J.-L. Merkens, J. Fang, A global analysis of subsidence, relative sea-level change and coastal flood exposure. *Nat. Clim. Change* **11**, 338–342 (2021).

47. R. Thiéblemont, G. Le Cozannet, R. J. Nicholls, J. Rohmer, G. Wöppelmann, D. Raucoules, Assessing current coastal subsidence at continental scale: Insights from Europe using the European Ground Motion Service. *Earth's Future* **12**, e2024EF004523 (2024).

48. Z. Yunjun, H. Fattahi, F. Amelung, Small baseline InSAR time series analysis: Unwrapping error correction and noise reduction. *Comput. Geosci.* **133**, 104331 (2019).

49. C. W. Chen, H. A. Zebker, Phase unwrapping for large SAR interferograms: Statistical segmentation and generalized network models. *IEEE Trans. Geosci. Remote Sens.* **40**, 1709–1719 (2002).

50. A. M. Guarnieri, S. Tebaldini, On the exploitation of target statistics for SAR interferometry applications. *IEEE Trans. Geosci. Remote Sens.* **46**, 3436–3443 (2008).

51. R. Jolivet, R. Grandin, C. Lasserre, M.-P. Doin, G. Peltzer, Systematic InSAR tropospheric phase delay corrections from global meteorological reanalysis data. *Geophys. Res. Lett.* **38**, 338–342 (2011).

52. H. P. Kierulf, H. Steffen, V. R. Barletta, M. Lidberg, J. Johansson, O. Kristiansen, L. Tarasov, A GNSS velocity field for geophysical applications in Fennoscandia. *J. Geodyn.* **146**, 101845 (2021).

53. G. Blewitt, D. Lavallée, Effect of annual signals on geodetic velocity. *J. Geophys. Res. Solid Earth* **107**, ETG 9-1–ETG 9-11 (2002).

54. M. S. Bos, R. M. S. Fernandes, S. D. P. Williams, L. Bastos, Fast error analysis of continuous GNSS observations with missing data. *J. Geodyn.* **87**, 351–360 (2013).

55. J. Langbein, Y. Bock, High-rate real-time GPS network at Parkfield: Utility for detecting fault slip and seismic displacements. *Geophys. Res. Lett.* **31**, 2003GL019408 (2004).

56. O. L. Stephenson, Y.-K. Liu, Z. Yunjun, M. Simons, P. Rosen, X. Xu, The impact of plate motions on long-wavelength InSAR-derived velocity fields. *Geophys. Res. Lett.* **49**, e2022GL099835 (2022).

57. R. Steffen, J. Legrand, J. Ågren, H. Steffen, M. Lidberg, Hv-lsc-ex2: Velocity field interpolation using extended least-squares collocation. *J. Geodyn.* **96**, 15 (2022).

58. Z. Altamimi, L. Métivier, P. Rebischung, H. Rouby, X. Collilieux, ITRF2014 plate motion model. *Geophys. J. Int.* **209**, 1906–1912 (2017).

59. Z. Altamimi, P. Rebischung, L. Métivier, X. Collilieux, ITRF2014: A new release of the International Terrestrial Reference Frame modeling nonlinear station motions. *J. Geophys. Res. Solid Earth* **121**, 6109–6131 (2016).

60. NOAA Continuously Operating Reference Stations (CORS) Network (NCN), NOAA Continuously Operating Reference Stations (CORS) Network website and online data portal; https://geodesy.noaa.gov/CORS/.

61. Y. Bock, P. Fang, A. Knox, A. Sullivan, S. Jiang, K. Guns, D. Golriz, A. Moore, D. Argus, Z. Liu, S. Kedar, "Extended Solid Earth Science ESDR System (ES3): Algorithm theoretical basis document: Chapter 4.2 (2021); http://garner.ucsd.edu/pub/measuresESESES_products/ATBD/ESESES-ATBD.pdf.

62. T. A. Herring, T. I. Melbourne, M. H. Murray, M. A. Floyd, W. M. Szeliga, R. W. King, D. A. Phillips, C. M. Puskas, M. Santillan, L. Wang, Plate boundary observatory and related networks: GPS data analysis methods and geodetic products. *Rev. Geophysics* **54**, 759–808 (2016).

63. M. Heflin, A. Donnellan, J. Parker, G. Lyzenga, A. Moore, L. G. Ludwig, J. Rundle, J. Wang, M. Pierce, Automated estimation and tools to extract positions, velocities, breaks, and seasonal terms from daily GNSS measurements: Illuminating nonlinear Salton Trough deformation. *Earth Space Sci.* **7**, e2019EA000644 (2020).

64. M. E. Keogh, T. E. Tornqvist, Measuring rates of present-day relative sea-level rise in low-elevation coastal zones: A critical evaluation. *Ocean Sci.* **15**, 61–73 (2019).

65. S. J. Taylor, B. Letham, Forecasting at scale. *Am. Stat.* **72**, 37–45 (2018).

66. P. Bird, An updated digital model of plate boundaries. *Geochem. Geophys. Geosyst.* **4**, 10.1029/2001GC000252 (2003).

67. European Space Agency, Sinergise, Copernicus Global Digital Elevation Model (2021); https://doi.org/10.5270/ESA-c5d3d65.

68. B. C. O'Neill, C. Tebaldi, D. P. Vuuren, V. Eyring, P. Friedlingstein, G. Hurtt, R. Knutti, E. Kriegler, J.-F. Lamarque, J. Lowe, G. A. Meehl, R. Moss, K. Riahi, B. M. Sanderson, The scenario model intercomparison project (ScenarioMIP) for CMIP6. *Geosci. Model Dev.* **9**, 3461–3482 (2016).

69. G. Garner, T. H. Hermans, R. Kopp, A. Slangen, T. Edwards, A. Levermann, S. Nowicki, M. D. Palmer, C. Smith, B. Fox-Kemper, H. Hewitt, IPCC AR6 WGI sea level projections. *Tech. Rep.* (2024).

70. C. W. Jennings, W. A. Bryant, Fault activity map of California. *Calif. Geol. Surv. Geol. Data Map* **6**, (2010).

71. Y. Bock, D. Melgar, Physical applications of GPS geodesy: A review. *Rep. Prog. Phys.* **79**, 106801 (2016).

72. C. W. Johnson, N. Lau, A. Borsa, An assessment of global positioning system velocity uncertainty in California. *Earth Space Sci.* **8**, e2020EA001345 (2021).

73. L. Caron, E. R. Ivins, E. Larour, S. Adhikari, J. Nilsson, G. Blewitt, GIA model statistics for GRACE hydrology, cryosphere, and ocean science. *Geophys. Res. Lett.* **45**, 2203–2212 (2018).

74. T. A. Herring, R. W. King, S. C. McClusky, GAMIT Reference Manual. GPS Analysis at MIT. Release 10 (2006), pp. 1–82.

75. W. Bertiger, Y. Bar-Sever, A. Dorsey, B. Haines, N. Harvey, D. Hemberger, M. Heflin, W. Lu, M. Miller, A. W. Moore, D. Murphy, GipsyX/RTGx, a new tool set for space geodetic operations and research. *Adv. Space Res.* **66**, 469–489 (2020).

76. G. Blewitt, C. Kreemer, W. C. Hammond, J. Gazeaux, MIDAS robust trend estimator for accurate GPS station velocities without step detection. *J. Geophys. Res. Solid Earth* **121**, 2054–2068 (2016).

77. A. Santamaría-Gómez, J. Ray, Chameleonic noise in GPS position time series. *J. Geophys. Res. Solid Earth* **126**, e2020JB019541 (2021).
